# A robot-assisted imaging pipeline for tracking the growths of maize ear and silks in a high-throughput phenotyping platform

**DOI:** 10.1186/s13007-017-0246-7

**Published:** 2017-11-08

**Authors:** Nicolas Brichet, Christian Fournier, Olivier Turc, Olivier Strauss, Simon Artzet, Christophe Pradal, Claude Welcker, François Tardieu, Llorenç Cabrera-Bosquet

**Affiliations:** 1 LEPSE, INRA, Montpellier SupAgro, Univ Montpellier, Montpellier, France; 2 Inria, Virtual Plants, Montpellier, France; 3 LIRMM, Department of Robotics, Univ Montpellier, 34392 Montpellier, France; 4 CIRAD, UMR AGAP, 34398 Montpellier, France; 5 AGAP, Univ Montpellier, CIRAD, INRA, Inria, Montpellier SupAgro, Montpellier, France

**Keywords:** Image-assisted phenotyping, Computer vision, Robot-assisted imaging, Machine learning, Maize, Water deficit

## Abstract

**Background:**

In maize, silks are hundreds of filaments that simultaneously emerge from the ear for collecting pollen over a period of 1–7 days, which largely determines grain number especially under water deficit. Silk growth is a major trait for drought tolerance in maize, but its phenotyping is difficult at throughputs needed for genetic analyses.

**Results:**

We have developed a reproducible pipeline that follows ear and silk growths every day for hundreds of plants, based on an ear detection algorithm that drives a robotized camera for obtaining detailed images of ears and silks. We first select, among 12 whole-plant side views, those best suited for detecting ear position. Images are segmented, the stem pixels are labelled and the ear position is identified based on changes in width along the stem. A mobile camera is then automatically positioned in real time at 30 cm from the ear, for a detailed picture in which silks are identified based on texture and colour. This allows analysis of the time course of ear and silk growths of thousands of plants. The pipeline was tested on a panel of 60 maize hybrids in the PHENOARCH phenotyping platform. Over 360 plants, ear position was correctly estimated in 86% of cases, before it could be visually assessed. Silk growth rate, estimated on all plants, decreased with time consistent with literature. The pipeline allowed clear identification of the effects of genotypes and water deficit on the rate and duration of silk growth.

**Conclusions:**

The pipeline presented here, which combines computer vision, machine learning and robotics, provides a powerful tool for large-scale genetic analyses of the control of reproductive growth to changes in environmental conditions in a non-invasive and automatized way. It is available as Open Source software in the OpenAlea platform.

**Electronic supplementary material:**

The online version of this article (10.1186/s13007-017-0246-7) contains supplementary material, which is available to authorized users.

## Background

Maize (*Zea mays* L.) silks are styles emerging from modified leaf sheaths (husks) that enclose the ear. Silk emergence and growth largely determine the final number of ovaries that develop into grains [1–3]. This is of particular importance under water deficit, because grain abortion is largely controlled in this case by the time during which silks elongate outside the husks. This time can range from 1 day in water deficit, associated with abortion rates of 70–90%, to 7 days in well-watered plants with low abortion rate [1, 4, 5]. The drought-dependent abortion rate is one of the main causes of the high sensitivity of maize to water deficit, so a precise characterization of silk growth and of its response to water deficit is crucial for estimating the degree of sensitivity of maize varieties to water deficit.

Silk number and growth have been measured by cutting cross sections of the silk bundle emerged from husks, and counting and measuring silk segments by image analysis [6–8]. This method is invasive and laborious because silks need to be sampled daily (up to 15 min per sample) [9]. It can also be followed with displacement transducers, thereby providing precise measurements of silk growth dynamics and its response to water deficit [10, 11]. This method is time-consuming and can be performed on a few tens of plants at maximum. Hence, current methods provide accurate estimates of silk number and growth but cannot be used at the throughput required for genetic analyses.

Phenotyping platforms based on computer vision are powerful tools for capturing at high-throughput a number of traits related with the structure and function of plants [12–14] such as the detection, count and quantification of morphological features of oat inflorescences [15], maize tassels [16, 17] and rice panicles [18]. Most imaging methods are based on camera viewpoints at fixed positions. This limits the possibilities for extracting complete information from complex images, and thus requires manual selection of best views containing useful information [12, 19, 20]. This is the case for maize ears whose position along the stem differs among genotypes and treatments, and is often hidden by leaves. Three problems need to be solved for automating image analysis of ear and silk growth, namely (1) detecting the position of the ear along the stem before the ear is visible (a non-intuitive detection that requires skills of maize experts) (2) identifying the best viewpoints for capturing silk growth dynamics and (3) following silk growth during the 1–7 days during which silks elongate outside the husks. Robot-assisted imaging may help solving these three problems by establishing a loop between image acquisition, analysis and de novo positioning of sensors [12, 21–23]. Thus, partial information recovered from an initial set of fixed viewpoints can be used to calculate new viewpoints containing maximum information and to guide a robot to acquire new images.

In this paper, we have combined computer vision methods, machine learning and robotics to develop a non-invasive, reproducible, and automatized pipeline for detecting maize ears and silks and monitoring silk growth dynamics in a high-throughput phenotyping platform. The methods presented here were tested in a panel of 60 maize genotypes subjected to different water availabilities in the PHENOARCH phenotyping platform (http://bioweb.supagro.inra.fr/phenoarch).

## Methods

The pipeline presented here involved six steps, namely (1) multi-view whole plant acquisition, (2) image segmentation, (3) detection of side view images containing maximum information, (4) detection of potential ear position, (5) robot-assisted movement of a camera near the ear and (6) ear and silk image acquisition and analysis.

### Step 1: Multi-view whole plant acquisition

RGB colour images (2056 × 2454 pixels) of each plant were taken daily with thirteen views (twelve side views from 30° rotational difference and one top view) by using the imaging units of the PHENOARCH platform [24]. Each unit is composed of a cabin involving top and side RGB cameras (Grasshopper3, Point Grey Research, Richmond, BC, Canada) equipped with 12.5–75 mm TV zoom lens (Pentax, Ricoh Imaging, France) and LED illumination (5050–6500 K colour temperature). Images were captured while the plant was rotating at constant rate (20 rpm) using a brushless motor (Rexroth, Germany).

### Step 2: Image segmentation

**Fig. 1:**
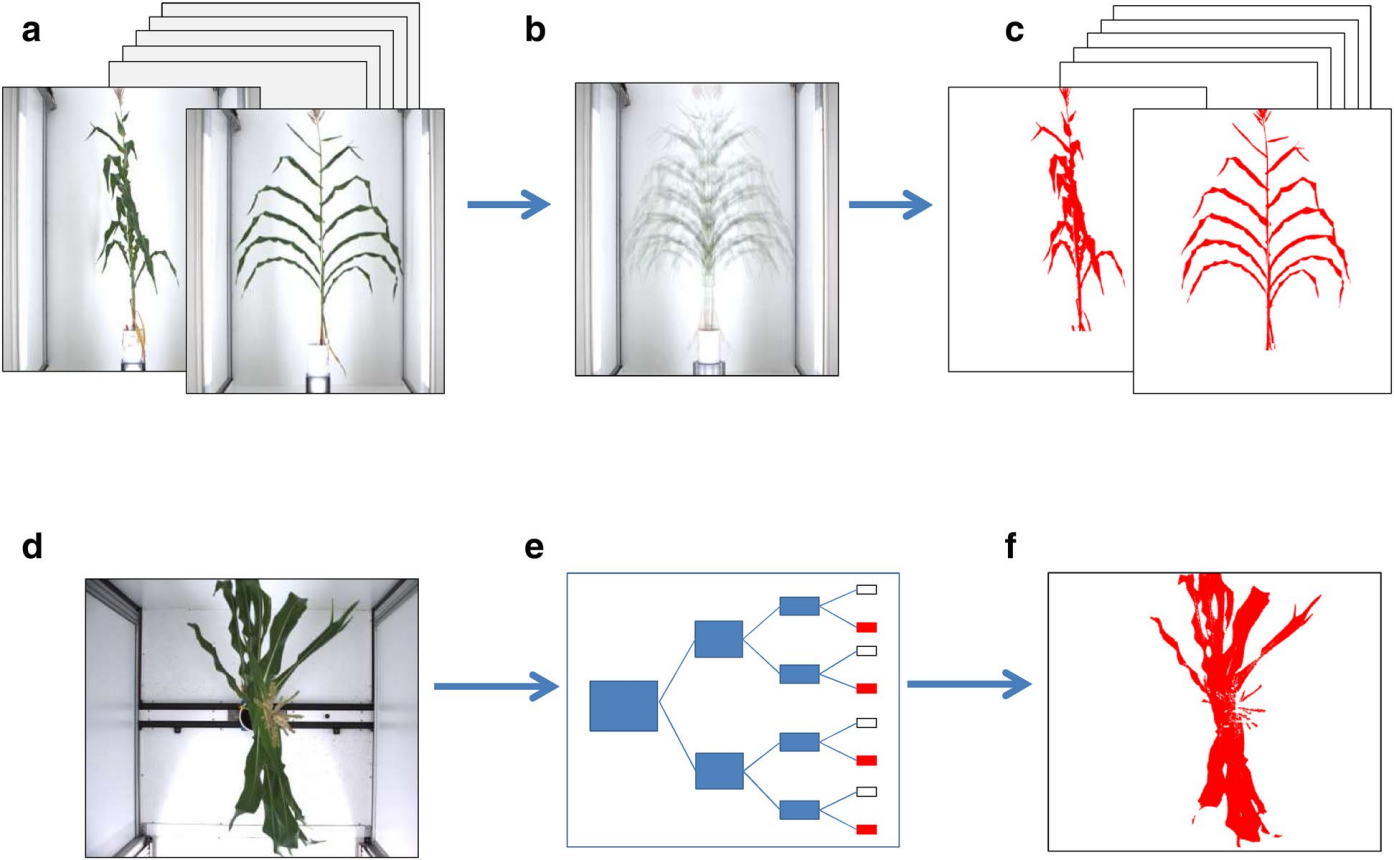
Segmentation procedures for extracting plant pixels from side and top view images. **a** Twelve side view images were obtained per plant and day with 30° angles between them, **b** mean image, **c** resulting segmented side view images, **d** top view image, **e** decision tree learning based on RGB, HSV, Luv, Lab, HLS, xyz, Yuv colour space, **f** resulting segmented top image

For side view images, pixels corresponding to the plant were segmented from those of the background by combing two threshold algorithms. This was performed by using a ‘mean shift’ method [25, 26] and a thresholding using Python [27]/OpenCV libraries (Open Source Computer Vision Library, http://opencv.org). In a first step, a mean image was calculated for each plant and day by pooling the twelve side view images acquired each 30° (Fig. 1a, b). The values of red, green and blue intensities in each pixel of each individual side image were then divided by those corresponding to pixels of the mean image (Fig. 1b) and a threshold was applied on the result of this division to extract plant pixels. In a second step, a HSV threshold algorithm [28] on pre-defined bound values (i.e. green and brown) was performed to retrieve some plant pixels that could have disappeared during the first step (Fig. 1c).

For top view images (Fig. 1d), plant pixels were segmented from background (Fig. 1f) using a decision tree learning [29] with seven colour space (RGB, HSV, Luv, Lab, HLS, xyz, Yuv) (Fig. 1e) implemented in R [30]. Learning was previously built on a set of contrasting top images involving plants of different genotypes and growth stages.

### Step 3: Selection of side view images containing maximum information

**Fig. 2:**
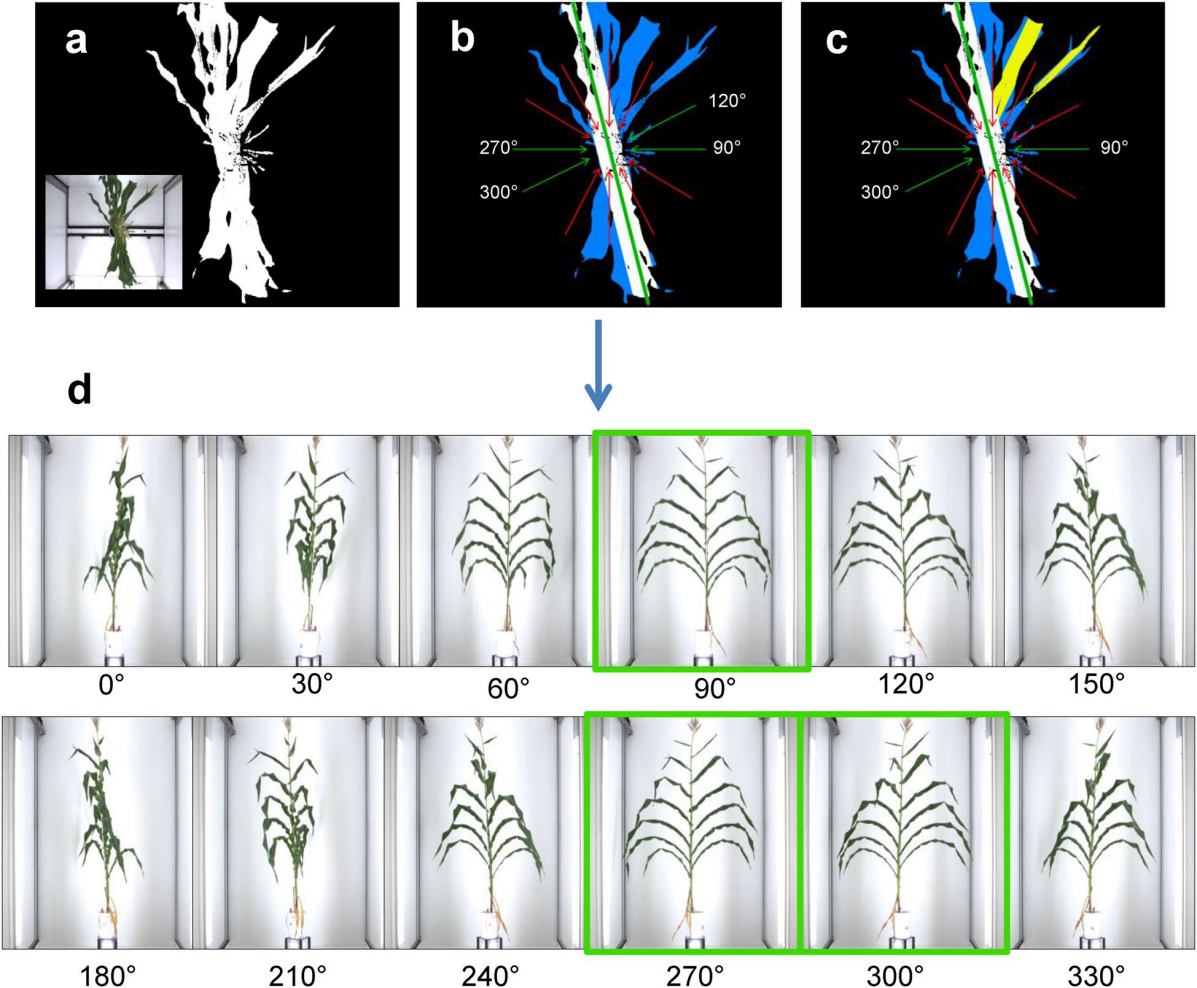
Detection of best side view images based on plant exemplified in Fig. 1. **a** Segmented and RGB (inset) top view images, **b** first robust major axis regression for identification of the direction of the plane carrying most leaves (green line), useful pixels group for this calculation (white pixels) and useless pixels group (blue pixels); useful and non-useful side view angles (green and red arrows, respectively), **c** rejected pixels after second robust reduced major axis regression on useless pixels group (yellow pixels), and interesting and non-useful side view angles (green and red arrows, respectively), **d** selected side view images containing most information

Maize leaves are essentially located in one plane (Fig. 1a, d) so side view images are associated with different degrees of occlusion of the stem and ear by leaves (Fig. 1a). The best side view images containing most information for detecting the ear position were chosen for each plant and day as those where the stem was most visible, perpendicularly to the plane containing leaves (Fig. 1a). To that end, we first used the segmented top view image of the plant (Fig. 2a) on which we performed a robust reduced major axis regression that limits the influence of outliers [31]. This step allowed identification of the orientation of the plane containing most leaves (Fig. 2b, green line) and of useful and useless pixel groups for this calculation (Fig. 2b, white and blue pixels, respectively). We have in this way identified the side view images containing maximum and minimum information (green and red arrows, respectively, Fig. 2b). In a second step, we performed a second robust reduced major axis regression on pixels group rejected by the first step in order to detect leaves that may hamper stem detection (Fig. 2c, yellow pixels). Overall, this allowed selecting from one to six side views per plant (green arrows, Fig. 2c) where the stem was the most visible (images highlighted in green, Fig. 2d) and discarding those where the stem was not visible.

### Step 4: Detection of the most likely position of the ear in selected side view images

**Fig. 3:**
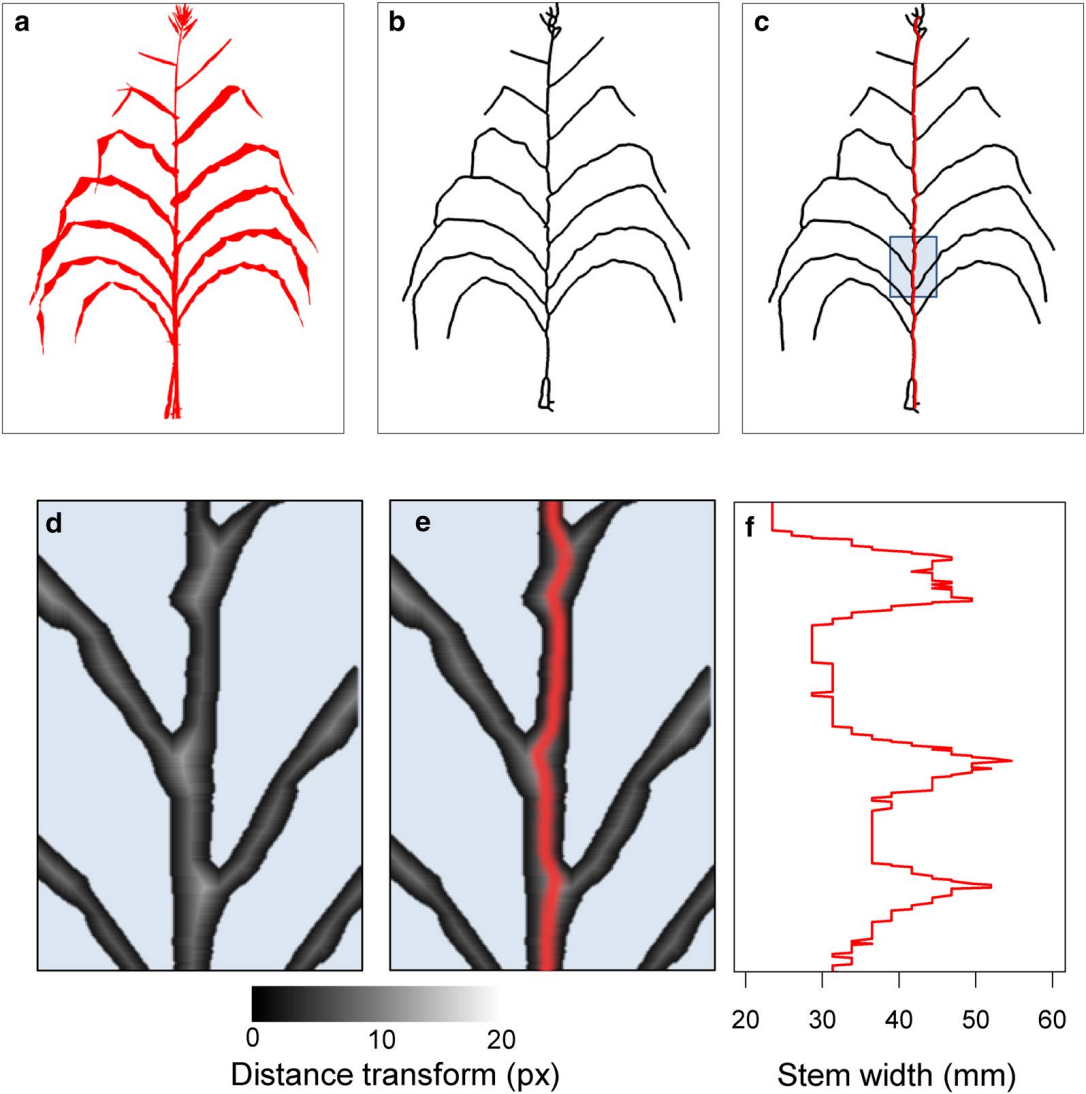
Stem detection procedure and calculation of stem width. **a** Side view segmented image based on plant exemplified in Fig. 1, **b** plant skeleton extracted from the segmented image, **c** extracted plant stem from the skeleton computed as the shortest path between the lowest and highest nodes on the skeleton (red line), **d** distance transform algorithm on segmented image (zoomed area shown in **c**) representing for each pixel of the image the distance to the nearest boundary pixel, **e** superimposed distance transform image to the stem extract from plant skeleton and **f** stem width along the stem, including the width at each leaf starting point

The apparent width of stem internodes (as observed on images, independently of the real width as measured after dissection) appreciably varies around the ear position. The ear being the last initiated lateral axis [32], internodes located above the ear are much thinner than those at the base of the stem. Second, due to ear development itself, the internode carrying the ear appears as slightly wider than those located below and above it. We have used these two criteria to estimate the most likely position of the ear in each image. The first step consisted in detecting the stem, by using the skeletons of selected side view images (Fig. 3a) extracted with the medial axis algorithm of ‘scikit-image’ library [33]. The resulting skeleton (Fig. 3b) was then analysed as a graph to extract the shortest path between the plant lowest node, located at the top centre of the pot in each side view image, and its highest node located at the highest leaf junction. We used for that a ‘shortest path’ algorithm [34] (Fig. 3c). The second step consisted in measuring stem width as a function of position along the stem. We first applied a distance transform algorithm from Python/OpenCV [35] on segmented images. We computed in this way, for each pixel of the image, the distance to the nearest boundary pixel (Fig. 3d). This image was then superimposed onto the extracted stem skeleton (Fig. 3e). The stem width (in pixels) was then determined along the stem skeleton as two times the corresponding distance transform pixel value (Fig. 3e). The resulting ‘width curve’ presented alternations of low values corresponding to internodes and of peaks corresponding to leaf starting points (Fig. 3f).

Knowing that the primary ear is located in the upper half of the stem [32] we have estimated a reference internode width in the lower half of the stem and use it to detect the position of thinner internodes in the upper half. To that end, we have ordered width values in the lower half and kept the 15% percentile, thereby eliminating artefactual width peaks corresponding to leaf junctions, to leaves occulting the stem and to errors related to the image of the plant tutor (Additional file 1). The most likely position of the ear was then estimated in the upper half by detecting (1) long peaks, presumably corresponding to the ear position, followed by (2) internodes with significantly lower width than the width in the lower half of the stem. Because artefacts such as wide leaf junctions or broken leaves along the stem may affect apparent internode width, the two criteria were weighed by 1 and 2, respectively. In cases where multiple side view images were selected, the last step consisted in keeping most represented positions by iteratively discarding those with the highest deviation.

### Step 5: Moving a camera close to the ear

**Fig. 4:**
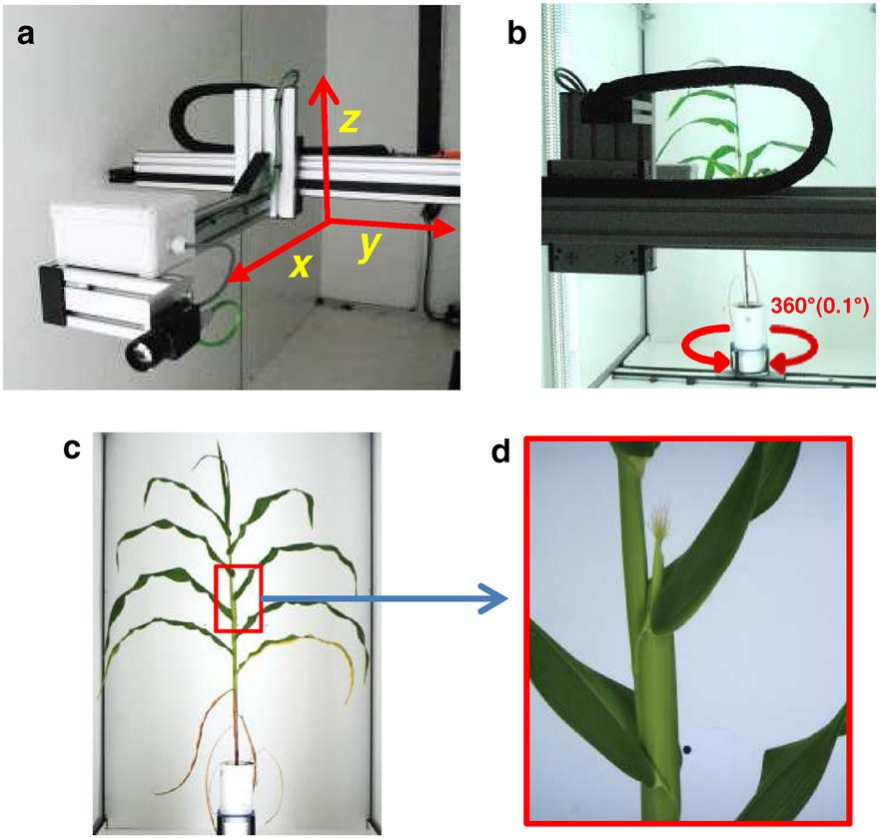
Robot-assisted imaging system for detailed ear and silk image acquisition. **a** Mobile camera installed in the imaging cabin equipped with a robotized arm able to move in the x, y, z directions, **b** brushless motor system allowing positioning the plant in such a way that the plane containing leaves is orthogonal to the camera axis, **c** side view image highlighting the selected region of interest and **d** detailed image at chosen x, y, z coordinates using the mobile camera

The procedure of image acquisition changed after an ear was detected on the studied plant. It was based on the successive use of two contiguous imaging cabins. Images of whole plants were acquired three times per day in the first cabin via the methods presented above. Selected pixel positions corresponding to the ear in side view images were transformed into [x, y, z] coordinates (Fig. 4a). This was performed using intrinsic and extrinsic camera matrices obtained with cameras calibration based on chessboard images (Additional file 2). The calibration was done with a minimization algorithm of a classic pinhole camera model [36], coupled with a turntable target chessboard model, using chessboard’s corners positions, acquired with OpenCV’s functions ‘findChessboardCorners’ and ‘cornerSubPix’. Imaging of the whole plant in the first imaging cabin took 20 s. Plants then moved to the second cabin while the system performed steps 2, 3, 4 and calculations of the [x, y, z] coordinates corresponding to the ear position, a process that took 90 ± 10 s. The process was even faster if [x, y, z] coordinates were manually validated and input in the system based on images of the former day.

In the second cabin, plants were automatically oriented in such a way that the plane containing leaves (as identified in step 3) was perpendicular to the camera axis, by using the brushless motor (Rexroth, Germany) that allows rotating plants with a precision of 0.1° (Fig. 4b). A robotized arm carrying the camera (Fig. 4a) was automatically positioned at 30 cm from the ear (Fig. 4c). This movement was driven by a linear profile axis able to move in the x, y, z directions (1500, 1000, 4000 mm moving range, respectively) equipped with electric synchronous servomotors (Rexroth, Germany) (Fig. 4a). Ears and silks were imaged by using a RGB camera (Grasshopper3, Point Grey Research, Richmond, BC, Canada equipped with a C-mount 50 mm fixed focal lens, Computar, CBC Group, USA) carried by the arm (Fig. 4d).

### Step 6: Analysis of ear and silk images

RGB images (2048 × 2448 pixels) of ears and silks (Fig. 4d) were then analysed using two different methods. First, total pixels corresponding to the plant were extracted from background by applying a thresholding based on HSV colour space. In a second method, pixels corresponding to silks were extracted from the previous step using a random forest classification method based on colour (Gaussian smoothing of 5 px) and texture (structure tensor eigenvalues of 1.6 px) (Ilastik software, version 1.1.7) [37]. We used for that a machine learning procedure based on a training set of contrasting ear images involving plants of different genotypes at different ear and silk developmental stages (Additional file 3). Finally, the time courses of pixels corresponding to silk bundles were individually fitted for each plant using the R scripts [30] ‘segmented’ package [38], and the maximum rates of silk growth and duration of silk growth were extracted.

#### Plant material, growth conditions and measured traits

The methods presented here were tested in an experiment involving a set of 60 commercial maize hybrids representative of breeding history in Europe during the last 60 years. This material covers a wide range of plant architecture, growth and development, leading to an appreciable variability of performances in the field. The experiment was conducted in the PHENOARCH phenotyping platform hosted at the M3P, Montpellier Plant Phenotyping Platforms (https://www6.montpellier.inra.fr/lepse/M3P), which allows non-destructive measurements of plant architecture and growth via automatic image acquisition (see [24] for platform details). Plants were sown in 9L pots filled with a 30:70 (v/v) mixture of a clay and organic compost. They were grown until 10 days after silk emergence. Two levels of soil water content were imposed; (1) retention capacity (WW, soil water potential of − 0.05 MPa) and (2) water deficit (WD, soil water potential of − 0.3 MPa). Soil water content in pots was maintained at target values by compensating transpired water three times per day via individual measurements of each plant. Each genotype was replicated 3 times. Greenhouse temperature was maintained at 25 ± 3 °C during the day and 20 °C during the night. Supplemental light (150 µmol m^−2^ s^−1^) was provided to extend the photoperiod to 16 h per day, and during day time when solar radiation dropped below 300 W m^−2^ (400 W HPS Plantastar lamps, OSRAM, Munich, Germany). Micro-meteorological conditions were monitored every 15 min at eight positions in the greenhouse at the top of the plant canopy.

Phenological stages, including anthesis and silk appearance were individually scored for each plant in the platform. All phenotypic, experimental and environmental collected data were stored in the PHIS information system (http://web.supagro.inra.fr/phis/web/index.php).

### Statistical analyses

Two-way analyses of variance (ANOVA) were performed using the ‘lm’ procedure to calculate the effects of water treatment and genotype. All statistical tests and graphs were performed using R 3.1.3 [30].

## Results and discussion

### Plant segmentation

**Fig. 5:**
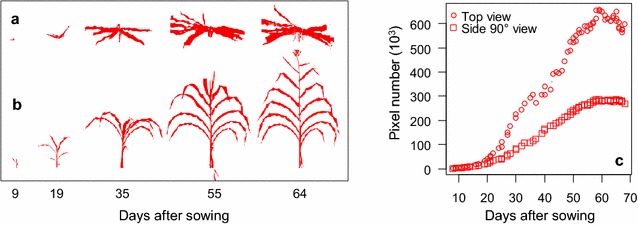
Plant representations at different development stages (1–8 weeks after sowing) for top (**a**) and side (**b**) images. **c** Time courses of the number of pixels corresponding to plants extracted from side and top views

The segmentation procedure for extracting plant pixels from side and top images proved efficient in all genotypes at all development stages (Fig. 5a, b; Additional file 4). The mean shift method was able to retrieve a maximum number of plant pixels, even in images where light exposure or plant colour changed (Additional file 5). Similarly, the tree learning method applied to top images provided a fast and precise segmentation. This pipeline was compatible with the routine management of experiments in the platform, which already involved acquisition of 13 images per plant and day. Thus, the first step of the pipeline provided accurate plant representations including stems, leaves and reproductive organs (ears and tassels) in addition of the routine estimations plant leaf area and bio-volume over time (Fig. 5c). Compared to other methods based on image cropping and different thresholding procedures that are species and platform dependent [39–42], the procedure presented here, based on the mean shift/tree learning method, has a wider application ability on multiple species and platforms with nearly no adjustment. Indeed, it has been successfully used in wheat, barley, tomato, cotton, grapevine [43–45], apple trees [46] and sorghum in other experiments and platforms.

### Selection of side images containing maximum information

The first step for selecting best side view images containing most information, i.e. a maximum number of leaves visible on the image (Fig. 2b), detected a high number of images per plant and day (4 and 6 out of 12 in 32 and 68% of cases, respectively, Table 1). The second step, that performs a second major axis regression on discarded pixels on top images (Fig. 2c), reduced this number by only keeping images where the stem was the most visible and with most leaves growing in the main plane (Fig. 2d). This step decreased the number of selected side images per plant and day to four or less in 59% of cases (Table 1). Selected images were, in 80% of cases, oriented in planes at 90° and 270° in relation to the plant row. This preferential across-row orientation of leaves is caused by the presence of neighbouring plants (low red to far-red light ratios), that results in a reduction of mutual shading and thus competition for light [47, 48].

**Table 1:** Number of side view images selected for detecting ear positions, based on the successive use of two robust major axis regressions (step 3)

Selected images	1st regression (%)	2nd regression (%)
1	–	2.0
2	–	9.1
3	–	15.2
4	32.1	32.3
5	–	17.3
6	67.9	24.1

### Detection of most likely position of the ear in each side view image

**Fig. 6:**
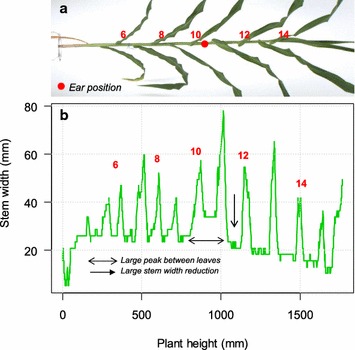
An example of procedure for extracting ear position. **a** Side view image of the plant. Numbers correspond to leaf ranks and red point to the ‘ground truth’ ear position, **b** stem width along the stem, with alternations of low values corresponding to internodes and of peaks corresponding to leaves (numbered in red). The assumed ear position corresponds to (i) a wider internode (between leaves 10 and 11, horizontal arrow) and (ii) a clear decrease in internode width from internode 11 onwards (vertical arrow)

The method presented here used several side view images to avoid possible errors that may occur during the different steps of the image analysis. For instance, the detection of the top of the stem was sometimes affected by leaves crossing each other. The major axis regression allowed detecting such errors. A low value of *R* ^2^ (< 0.82) indicated that the ‘stem’ detected by the algorithm did not correspond to the real stem. The corresponding side view image was discarded in this case (Additional file 6). The detection of plant skeletons combined with distance transform algorithm (Fig. 3) in selected side images allowed identification of stems and accurate estimation of stem width (Fig. 6).

The ‘stem width’ curves corresponding to all studied plants consisted in alternations of low values corresponding to internodes and of peaks corresponding to leaves (numbered in red in Fig. 6b). Stem width in the lower half of the plant (nodes 5–9) could be identified with acceptable accuracy (20.6 ± 4.8 mm) in 85% of plants. This was the cases when this reference stem width was calculated based on the 15% percentile of values, whereas using mean values resulted in a 40% of erroneous values. In the plant represented in Fig. 6, the wider internode between leaves 10 and 11 corresponded to the ear (first criterion for ear detection). This was consistent with the second criterion, a clear decrease in internode width from internode 11 onwards (vertical arrow, Fig. 6b). This analysis was performed for each selected side image of each plant to estimate potential ear positions (red dot, Fig. 6a).

The method was applied in a panel of 60 maize hybrids subjected to either well-watered or water deficit conditions. Over 360 plants, primary ear position was correctly estimated in 86% of cases on the day when the first silk appeared, at a time before ears could be visually assessed (Table 2). This was already the case in half of cases 2 days before the first silk appearance, a performance close to that of best maize experts. Hence, the method could be considered as successful in spite of the morphological differences between genotypes and the differences in growth caused by water deficit. In the 14% unsuccessful cases, the ear was detected several times before flowering but not the day of silking in 77% of cases, resulting in a minor problem if the history of each plant was tracked over days and input to the system. In the remaining unsuccessful cases (23%, 12 plants), the ear was not detected because the image pipeline analysis failed due to a distribution of leaves that did not follow a plane, thereby impeding one to extract good skeletons and to estimate reliable stem widths (Additional file 7). This problem may be overcome by using a 3D plant reconstruction [24, 49]. However the latter method requires more computing time compared with the method presented here. Because a real-time procedure is essential for high throughput, we have preferred the option of tagging the unsuccessful plants so the operator can visually inspect them and input his or her best guessed position into the system for further analyses.

**Table 2:** Percentages of ear position correctly detected as a function of days before the appearance of silks

Days before silking	WW (%)	WD (%)
0	83	88
1	65	69
2	51	49
3	33	28
4	18	17
5	7	6

WW and WD refer to well-watered and water-deficit treatments, respectively. N = 360 plants

### XYZ positioning of a mobile camera and image acquisition

**Fig. 7:**
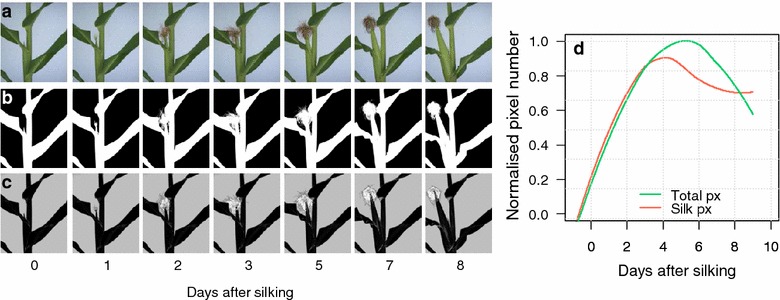
Analysis of detailed images of ears and silks. **a** Sequential images over 8 days after silking, **b** segmented ear images including all plant parts (white pixels), **c** segmented images based on colour and texture segmentation allowing extraction of pixels corresponding to silks (white pixels), **d** time course of the normalised extracted pixels numbers corresponding to leaves, ear and silks (green line) or to silks (red line)

The robot arm driven by the synchronous servomotors allowed a highly precise positioning of the moving camera close to the ear with an error of less than 0.1 mm. This allowed us to acquire high quality and repeatable images of the growing ear over time (Fig. 7a; see video in Additional file 8). The positioning of the camera and the ear image acquisition was fast enough allowing phenotyping hundreds of plants several times per day. Similar works have combined computer vision methods and robotics to increase the reconstruction accuracy of highly occluded complex plant structures [21, 50, 51]. Here, the combination of these methods allowed establishing a loop between extraction of partial descriptions of side view images and the positioning of the mobile camera, and thus acquiring precise images of ears and silks.

### Dynamic monitoring of silk growth traits and differences between genotypes and water treatments

The imaging procedure presented above resulted in ear images with a high spatial and temporal resolution, and allowed us to monitor silk growth dynamics from silk emergence until silk senescence in primary ears (Fig. 7a; see video in Additional file 8).

**Fig. 8:**
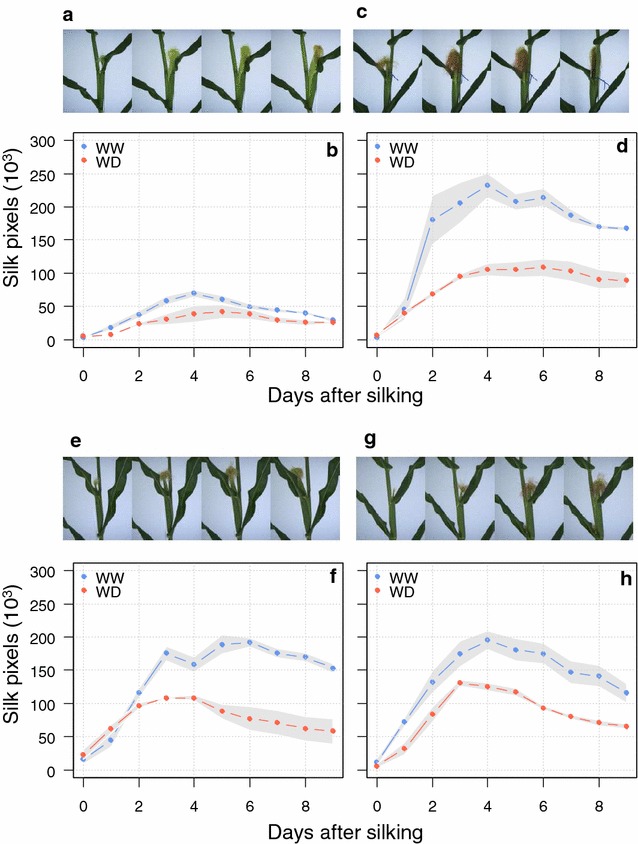
Time course of silk growth rate (SGR) in four hybrids with markedly different leaf architectures and colours of silks under well-watered (WW) and water deficit (WD) conditions. **a**, **c**, **e**, **g** detailed images of ears and silks; **b**, **d**, **f**, **h** silk growth rate over 7 days after silking. Points, mean of three replicates, shaded areas, standard error

Image analysis based on colour and texture allowed proper identification of silks compared to the background, leaves, ear and husks (white pixels, Fig. 7c). This was not the case with simple colour segmentation (Fig. 7b). The resulting time course of silk growth (red line, Fig. 7d) differed from that of the sum of total ear, husks and silk growth (green line, Fig. 7d). The segmentation procedure remained valid for silks with different colour and shape, as shown in Fig. 8 for four lines presenting markedly different colours (Fig. 8a, c, e, g). The time course of silk growth was described with sufficient precision to clearly identify the differences between genotypes and between watering treatments (Fig. 8b, d, f, h). Silks growth rates were maximum just after silks emerged out of the husks and progressively decreased with time (Fig. 8b, d, f, h). Lower silk growth rates were observed in water deficit (Fig. 8b, d, f, h) as they were in precise measurements at low throughput [10, 11, 52].

The method was applied in 60 maize hybrids subjected to two watering regimes (Additional file 9). An analysis of variance (ANOVA) identified significant effects of genotypic differences and of water deficit on maximum silk growth rate (*P* < 0.001, Table 3). A significant genotypic effect was also observed for the duration of silk growth (*P* < 0.001, Table 3). Studies based on destructive measurements of silks have reported genotypic variation in silk growth rates and duration [1, 52, 53] as well as a decrease in these traits due to water deficit [1, 11].

**Table 3:** Output of an analysis of variance performed on maximum rates of silk growth and duration of silk growth

Trait	WW	WD	ANOVA		
			Scenario	Genotype	GxE
Silk growth rate (pixel day^−1^)	50,843	33,325	***	***	*
Silk growth duration (day)	4.1	4.2	ns	***	***

The effects of watering treatment, genotype and genotype × watering treatments are shown, together with mean values in well-watered (WW) and water deficit (WD) treatments

*ns* no significant

* *P* < 0.05; *** *P* < 0.001

## Conclusions

By combining computer vision methods and robotics, the pipeline presented here provides for the first time an automatic and non-invasive procedure for monitoring silk growth dynamics at high-throughput in a phenotyping platform. It automatically detected ear position and evaluated silk growth in a panel of maize genotypes with contrasting size and architectures. It therefore provides a powerful tool for large-scale genetic analyses of the control of reproductive growth to changes in environmental conditions in reproductive structures in a non-invasive and automatized way.

## Additional files

**Additional file 1.** An example of an artefact affecting the apparent internode width. (a) Segmented side view image showing a broken leaf in the lower half of the stem. Green line represents the detected stem. (b) Graph representing the apparent ‘stem width’.

**Additional file 2.** Detailed information of calculations of [x,y,z] coordinates including intrinsic and extrinsic matrices.

**Additional file 3.** R HTML notebook detailing the procedure for extracting pixels corresponding to silks from ear images, and for reproducing figures.

**Additional file 4.** R HTML notebook allowing reproducing Fig. 5.

**Additional file 5.** Example of the segmentation procedure on images differing in light exposure.

**Additional file 6.** Example of a discarded side view image during the stem reconstruction step based on major axis regression. (a) Side view plant image and (b) the corresponding detected stem. Red line represents the major axis regression.

**Additional file 7.** Example of an ear detection problem. (a) Side view image of a plant with distribution of leaves that did not follow a plane. (b) Corresponding segmented image with erroneous detection of the stem (green line). Red arrow, actual ear position.

**Additional file 8.** Time-lapse video representing the growth of the ear and silks of a maize plant from 4 days before silking until 9 days after silking.

**Additional file 9.** Silk growth dynamics of the 60 studied maize lines grown under well-watered (WW) and water deficit (WD) conditions. Points, mean of three replicates, shaded areas, standard error.
