# Supplementary figures and images for: A robot-assisted imaging pipeline for tracking the growths of maize ear and silks in a high-throughput phenotyping platform
Source: Plant Methods. 2017 Nov 8;13:96. doi: 10.1186/s13007-017-0246-7 (PMC5688816; doi:10.1186/s13007-017-0246-7)

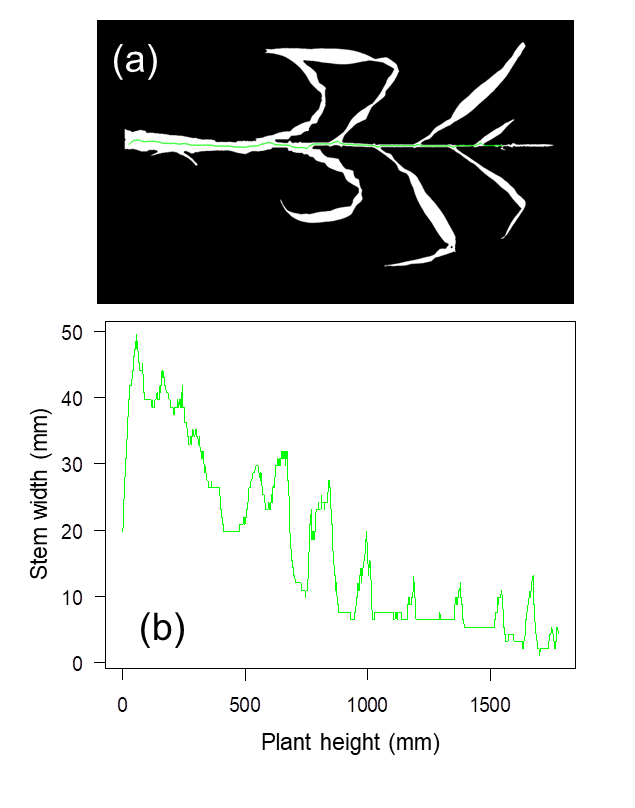

Supplement: Supplementary file 1 — Additional file 1. An example of an artefact affecting the apparent internode width. (a) Segmented side view image showing a broken leaf in the lower half of the stem. Green line represents the detected stem. (b) Graph representing the apparent ‘stem width’. [file 13007_2017_246_MOESM1_ESM.png]

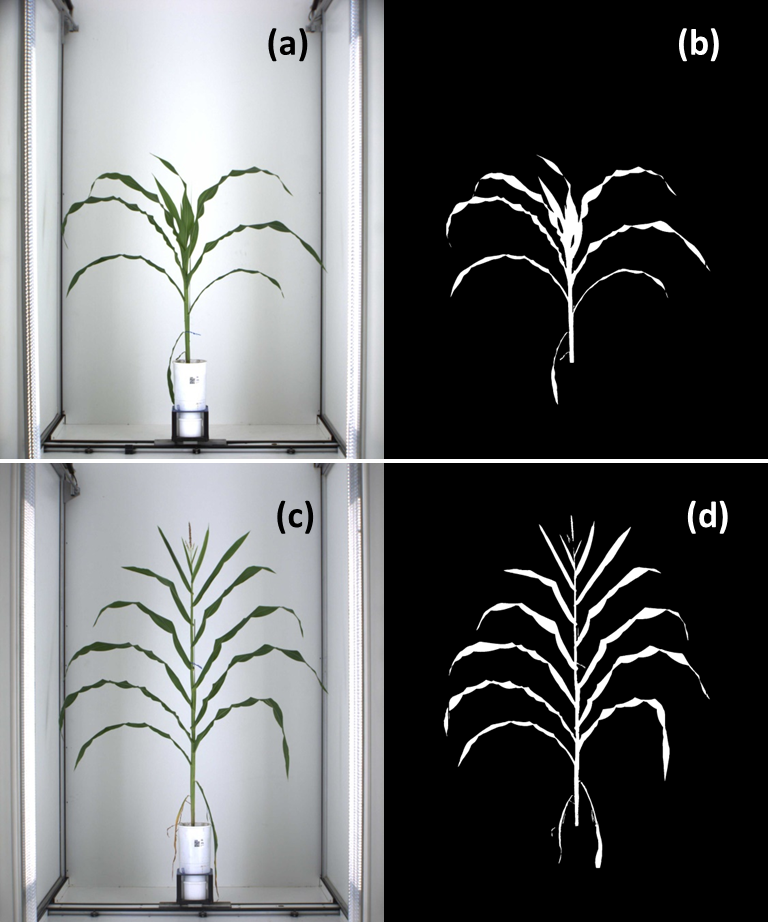

Supplement: Supplementary file 5 — Additional file 5. Example of the segmentation procedure on images differing in light exposure. [file 13007_2017_246_MOESM5_ESM.png]

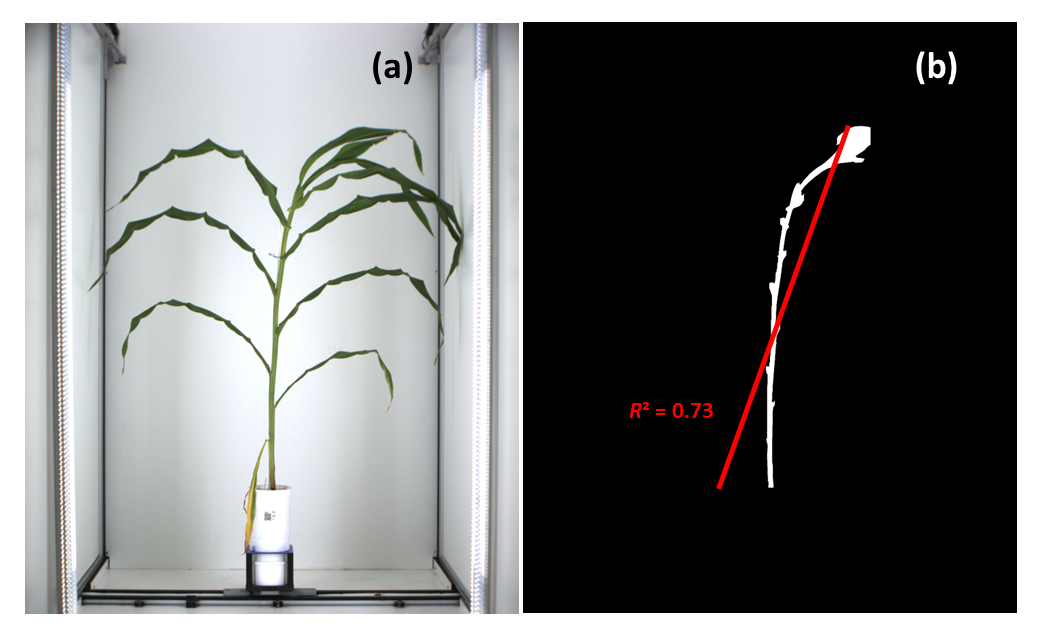

Supplement: Supplementary file 6 — Additional file 6. Example of a discarded side view image during the stem reconstruction step based on major axis regression. (a) Side view plant image and (b) the corresponding detected stem. Red line represents the major axis regression. [file 13007_2017_246_MOESM6_ESM.png]

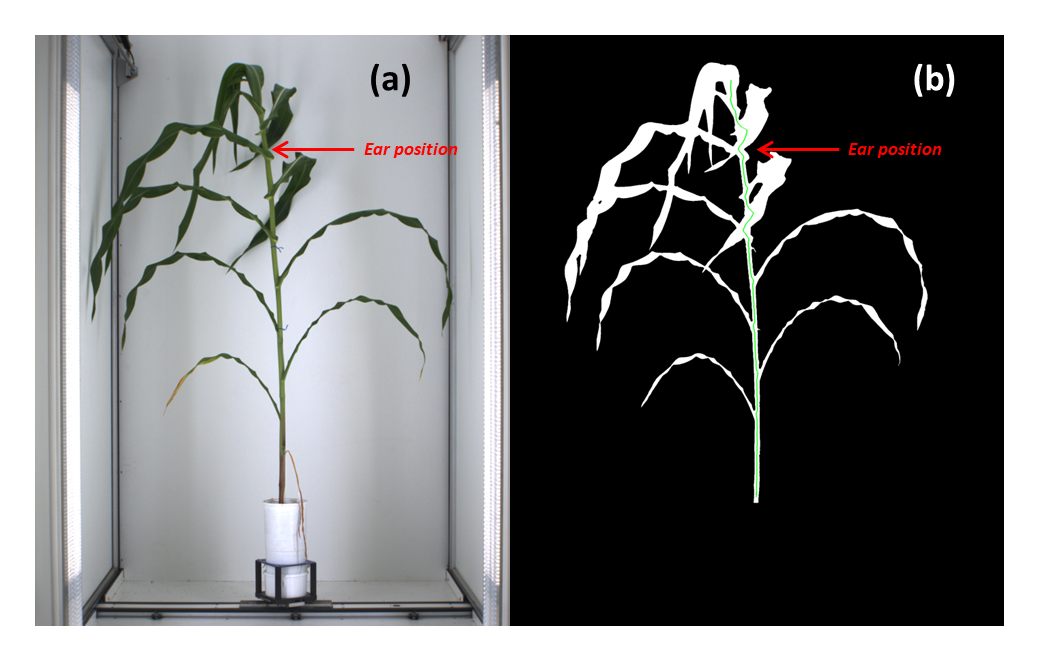

Supplement: Supplementary file 7 — Additional file 7. Example of an ear detection problem. (a) Side view image of a plant with distribution of leaves that did not follow a plane. (b) Corresponding segmented image with erroneous detection of the stem (green line). Red arrow, actual ear position. [file 13007_2017_246_MOESM7_ESM.png]
