# Supplementary material for: A robot-assisted imaging pipeline for tracking the growths of maize ear and silks in a high-throughput phenotyping platform
Source: Plant Methods. 2017 Nov 8;13:96. doi: 10.1186/s13007-017-0246-7 (PMC5688816; doi:10.1186/s13007-017-0246-7)
Supplement: Supplementary file 2 — Additional file 2. Detailed information of calculations of [x,y,z] coordinates including intrinsic and extrinsic matrices. [file 13007_2017_246_MOESM2_ESM.pdf]

The objective of motion of mobile camera (mounted on a robotic arm) is to focus on detected ear from images acquired with a fixed camera. We assume that intrinsic matrices and camera planes are the same for the computation. In a first step, mobile camera moves parallel to the camera plane in order to position the selected object (i.e. detected ear) in the center of the image. Then, we translate the camera in the plant direction to acquire an image of better resolution.

Let (u, v) be the pixel position in the image and (X, Y, Z) a 3D point in the world coordinate frame of the scene. The projection (u, v) of the point in the image is defined as:

$$s\begin{bmatrix}u\\v\\1\end{bmatrix}=\begin{bmatrix}f_x & 0 & c_x\\0 & f_y & c_y\\0 & 0 & 1\end{bmatrix}\begin{bmatrix}r_{11} & r_{12} & r_{13} & t_1\\r_{21} & r_{22} & r_{23} & t_2\\r_{31} & r_{32} & r_{33} & t_3\end{bmatrix}\begin{bmatrix}X\\Y\\Z\\1\end{bmatrix}$$

with (fx, fy) the focal length, (cx, cy) the center of the image in pixel, and R the transformation matrix between the camera frame and the global coordinate frame.

If we define the camera frame as the global coordinate frame, then the projection of a pixel (u1, v1) can be expressed as :

$$s\begin{bmatrix}u_1\\v_1\\1\end{bmatrix}=\begin{bmatrix}f_x & 0 & c_x\\0 & f_y & c_y\\0 & 0c & 1\end{bmatrix}\begin{bmatrix}1 & 0 & 0 & 0\\0 & 1 & 0 & 0\\0 & 0 & 1 & 0\end{bmatrix}\begin{bmatrix}X\\Y\\Z\\1\end{bmatrix} \Rightarrow \begin{bmatrix}su_1\\sv_1\\s\end{bmatrix}=\begin{bmatrix}Xf_x+Zc_x\\Yf_y+Zc_y\\Z\end{bmatrix}$$

X and Y object coordinates can be expressed as : $\begin{cases}X=\dfrac{Z(u_1-c_x)}{f_x}\\Y=\dfrac{Z(v_1-c_y)}{f_y}\end{cases}$ (1)

In the previous frame, we translate the camera frame to position the projection of (X,Y,Z) in the center of the image (u2,v2):

$$s\begin{bmatrix}u_2\\v_2\\1\end{bmatrix}=\begin{bmatrix}f_x & 0 & c_x\\0 & f_y & c_y\\0 & 0c & 1\end{bmatrix}\begin{bmatrix}1 & 0 & 0 & t_x\\0 & 1 & 0 & t_y\\0 & 0 & 1 & 0\end{bmatrix}\begin{bmatrix}X\\Y\\Z\\1\end{bmatrix} \quad \text{with} \quad \begin{cases}u_2=\dfrac{width}{2}\\v_2=\dfrac{height}{2}\end{cases}$$

X and Y object coordinates can be expressed as : $\begin{cases}X=\dfrac{Z(u_2-c_x)}{f_x}-t_x\\Y=\dfrac{Z(v_2-c_y)}{f_y}-t_y\end{cases}$ (2)

Combination of (1) and (2) gives the expression of t_x and t_y : $\begin{cases} t_x = \dfrac{Z(u_2 - u_1)}{f_x} \\ t_y = \dfrac{Z(v_2 - v_1)}{f_y} \end{cases}$

The extrinsic matrix is that it describes how the world is transformed relative to the camera, so the camera's target position is the opposite of calculated t_x and t_y.

The transformation matrix from fixed camera to mobile camera is a rotation matrix (fig. 1) :

$$\begin{bmatrix} X_a \\ Y_a \\ Z_a \end{bmatrix} = \begin{bmatrix} 0 & 0 & 1 \\ 1 & 0 & 0 \\ 0 & -1 & 0 \end{bmatrix} \begin{bmatrix} X_c \\ Y_c \\ Z_c \end{bmatrix} + \begin{bmatrix} X_0 \\ Y_0 \\ Z_0 \end{bmatrix} \Rightarrow \begin{bmatrix} X_a \\ Y_a \\ Z_a \end{bmatrix} = \begin{bmatrix} 0 & 0 & 1 \\ 1 & 0 & 0 \\ 0 & -1 & 0 \end{bmatrix} \begin{bmatrix} -t_x \\ -t_y \\ 0 \end{bmatrix} + \begin{bmatrix} X_0 \\ Y_0 \\ Z_0 \end{bmatrix}$$

The target position to pilot the mobile camera is finally :

$$\begin{cases} X_a = [depth, depending\ on\ desired\ resolution] + X_0 \\ Y_a = \dfrac{Z(u_1 - u_2)}{f_x} + Y_0 \\ Z_a = \dfrac{Z(v_2 - v_1)}{f_y} + Z_0 \end{cases}$$

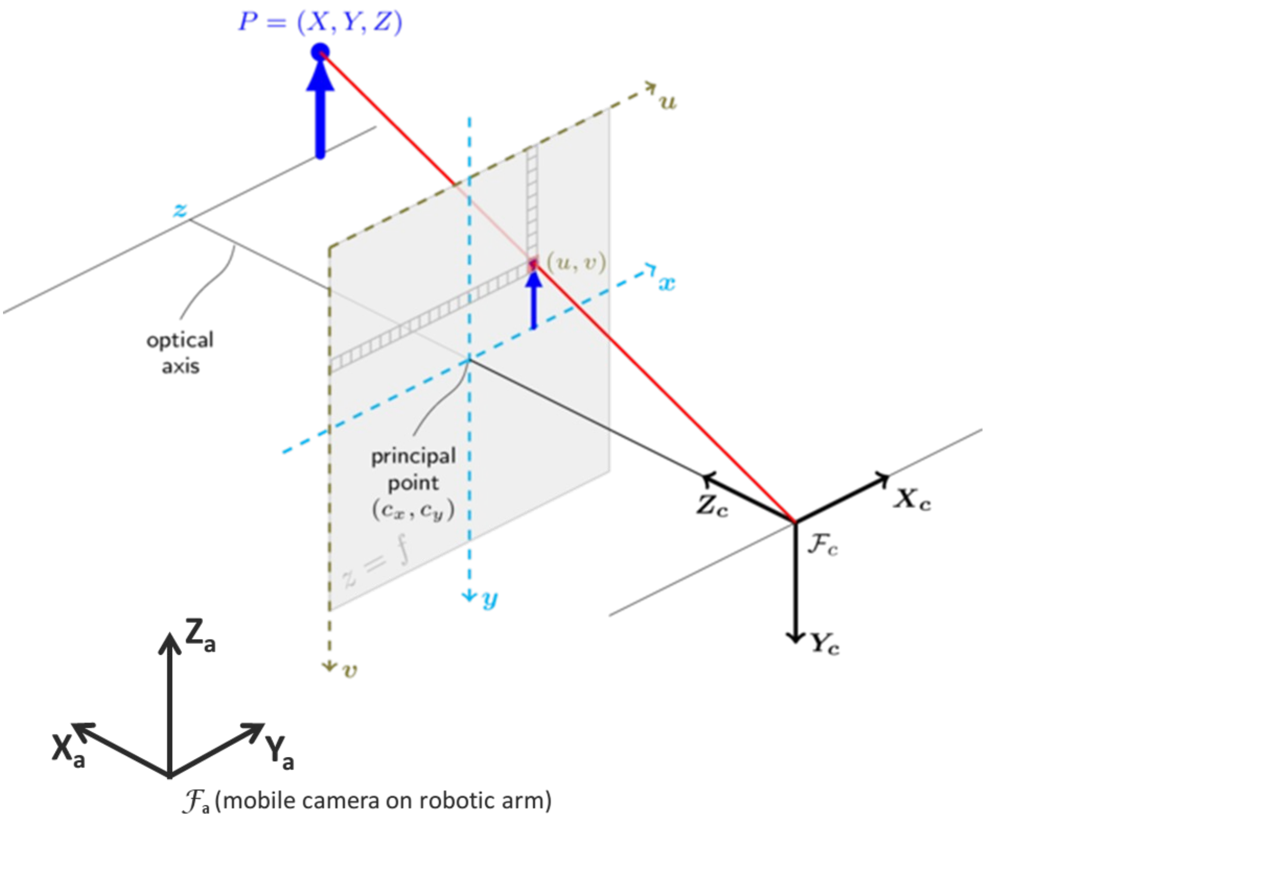

Adapted from http://docs.opencv.org/2.4/modules/calib3d/doc/camera_calibration_and_3d_reconstruction.html

Fig.1: Image, fixed camera and mobile camera coordinates in imaging cabin
