# Supplementary material for: A robot-assisted imaging pipeline for tracking the growths of maize ear and silks in a high-throughput phenotyping platform
Source: Plant Methods. 2017 Nov 8;13:96. doi: 10.1186/s13007-017-0246-7 (PMC5688816; doi:10.1186/s13007-017-0246-7)
Supplement: Supplementary file 3 — Additional file 3. R HTML notebook detailing the procedure for extracting pixels corresponding to silks from ear images, and for reproducing figures. [file 13007_2017_246_MOESM3_ESM.html]

Silk growth dynamics analysis


Code 

- Show All Code
- Hide All Code
- Download Rmd

# Silk growth dynamics analysis

#### *Nicolas Brichet & Llorenç Cabrera-Bosquet*


# Analysis of ear and silk images using Ilastik

Pixels corresponding to silks were extracted using a random forest classification method based on colour (Gaussian smoothing of 5 px) and texture (structure tensor eigenvalues of 1.6 px) in **Ilastik software, version 1.1.7**.

The following materials are available at  for reproducing silk extraction analysis.

A folder named DATASET SILKS contains:

1. `Training dataset`: image dataset of contrasting ear images involving plants of different genotypes at different ear and silk developmental stages
2. `Validation dataset`: image dataset of 3 well-watered and 3 water-deficit plants of the same genotype
3. `Ilastik project`: the file **silks\_classification\_light.ilp** contains all colour and texture classification features using the training image dataset
4. `Ilastik_output`: image dataset of Ilastik output resulting images of plant #316 present in the `Validation dataset`
5. `data_silk_ilastik_output.csv`: csv file containing silk pixels data of 6 plants

The pipeline is described in the following figure:

# Extract silk pixels from Ilastik outputs

This example shows how to extract silk pixels from Ilastik outputs


```
#Required R packages
if (!require("plyr")) install.packages("plyr")
if (!require("EBImage")) install.packages("EBImage")
```


```
par(mfrow=c(1,2))
colimg = readImage("./Training dataset/originals/310_2017-06-10_03-51-52_6855.png")
img = readImage("./Training dataset/ilastik_output/310_2017-06-10_03-51-52_6855_Probabilities.png")
display(colimg, method='raster')
display(img, method='raster')
```


```
img_thresh = img > .75 #apply threshold
computeFeatures.shape(img_thresh) #compute features
```


```
     s.area s.perimeter s.radius.mean s.radius.sd s.radius.min s.radius.max
TRUE 114619           5     0.8478015   0.2850134    0.4472136     1.264911
```

# Example: extract data from temporal series

This exemple shows how to extract silk pixels from a set of Ilastik output images corresponding to the plant #316


```
wd = "./Ilastik_output/"
file_list <- list.files(wd, pattern = ".png")
table_results <- NULL
for (file in file_list){
 
img = readImage(paste("./Ilastik_output/",file,sep="")) #read images
img_thresh = img > .75 #apply threshold
results <- cbind(file,computeFeatures.shape(img_thresh)) #compute features
table_results= as.data.frame(rbind(results,table_results))
 }
```

# Get data output from Ilastik

This exemple is based on a csv file that contains silk pixels from 6 plants corresponding to the same genotype


```
#Load csv file
data_output <- read.csv("./data_silk_ilastik_output.csv")
knitr::kable(head(data_output))
```


| plant | date | pixels\_number | Plant | Date | doy | flo | das | Scenario |
| --- | --- | --- | --- | --- | --- | --- | --- | --- |
| 1261 | 31/05/2017 00:36 | 6220 | 1261 | 31/05/2017 00:36 | 151 | 0 | 0.00 | WW |
| 1261 | 31/05/2017 18:07 | 48357 | 1261 | 31/05/2017 18:07 | 151 | 0 | 0.75 | WW |
| 1261 | 01/06/2017 00:37 | 70278 | 1261 | 01/06/2017 00:37 | 152 | 1 | 1.00 | WW |
| 1261 | 01/06/2017 18:41 | 152115 | 1261 | 01/06/2017 18:41 | 152 | 1 | 1.75 | WW |
| 1261 | 02/06/2017 00:34 | 161067 | 1261 | 02/06/2017 00:34 | 153 | 2 | 2.00 | WW |
| 1261 | 02/06/2017 18:43 | 211703 | 1261 | 02/06/2017 18:43 | 153 | 2 | 2.75 | WW |

# Compute slopes using segmented package

In this exemple time courses of pixels corresponding to silk bundles are individually fitted for each plant using the R package 1 ‘segmented’ 2, and the maximum rates of silk growth and duration of silk growth are extracted.


```
#Required R packages
if (!require("dplyr")) install.packages("dplyr")
if (!require("segmented")) install.packages("segmented")
if (!require("lubridate")) install.packages("lubridate")
```


```
#table for results
segmentedmethodtable=NULL
#For i loop
for (i in levels(factor(data_output$Plant))){
  
  segmentedplant = data_output[data_output$Plant==i,]
  
  x <-segmentedplant$das
  y <-segmentedplant$pixels_number
  
  p <- NULL
    try(p <- lm(y~x),silent=TRUE)
  l <- NULL
    try(l <- segmented(p, seg.Z =~x, psi = list(x=c(4))),silent=T)
  
  SER <- ifelse(is.null(l)==T,NA,slope(l)$x[1]) #silk expansion rate
  Duration <-ifelse(is.null(l)==T,NA,summary(l)$psi[2]) #silk growth duration (breakpoint)
  newline <- data.frame(ID= i, SER = as.numeric(as.character(SER)), Duration=as.numeric(as.character(Duration)))
  segmentedmethodtable <-rbind(segmentedmethodtable, newline)
  
}
segmentedmethodtable$Scenario <- data_output$Scenario[match(segmentedmethodtable$ID,data_output$plant)]
```

# Results


```
par(mfrow=c(1,2))
boxplot(segmentedmethodtable$SER~segmentedmethodtable$Scenario, col=c("tomato","cornflowerblue"), main= "Silk growth rate (pixel d-1)", las=1)
boxplot(segmentedmethodtable$Duration~segmentedmethodtable$Scenario, col=c("tomato","cornflowerblue"), main= "Silk growth duration (d)", las=1)
```

# Mean silk expansion rate and silk growth duration


| Scenario | SER | Duration |
| --- | --- | --- |
| WD | 48756.67 | 3.436890 |
| WW | 70380.00 | 3.500934 |

# Silk growth dynamics


```
par(bg=NA)
test = aggregate(data_output[c( "pixels_number")], by=data_output[c("flo","Scenario")], FUN=mean, na.rm=T)
test = test[test$flo>-3,] #limit to 3 days before flowering
testsd = aggregate(data_output[c("pixels_number")], by=data_output[c("flo","Scenario")], FUN=sd, na.rm=T)
testsd = testsd[testsd$flo>-3,] #limit to 3 days before flowering
#WW plants
kk = test[test$Scenario=="WW",]
kk$color = ifelse (kk$Scenario=="WW", "cornflowerblue", "tomato")
x= kk$flo
y= kk$pixels_number/1000
y.Err = na.omit(testsd[testsd$Scenario=="WW",]$pixels_number/1000)/ sqrt(nrow(testsd[testsd$Scenario=="WW",])-1)
y.Up = y + y.Err
y.Dn = y - y.Err
plot(x,y,xlim=c(0,9), ylim=c(0,300),xlab="Days after flowering", ylab="Silk pixels (10^3)", las=1, col='cornflowerblue',type='b', lwd=1.5, pch=20)
grid()
```


```
polygon(c(x,rev(x)),c(y.Up,rev(y.Dn)),col="grey90",border="white")
points(x,y,xlim=c(0,9), ylim=c(0,300),xlab="", ylab="", las=1, col='cornflowerblue',type='b', lwd=1.5, pch=20)
```


```
#WD plants
kk = test[test$Scenario=="WD",]
kk$color = ifelse (kk$Scenario=="WW", "cornflowerblue", "tomato")
x= kk$flo
y= kk$pixels_number/1000
y.Err = na.omit(testsd[testsd$Scenario=="WD",]$pixels_number/1000)/ sqrt(nrow(testsd[testsd$Scenario=="WD",])-1)
y.Up = y + y.Err
y.Dn = y - y.Err
points(x,y,xlim=c(0,9), ylim=c(0,300),xlab="", ylab="", las=1, col='cornflowerblue',type='b', lwd=1.5, pch=20)
polygon(c(x,rev(x)),c(y.Up,rev(y.Dn)),col="grey90",border="white", add=T)
```


```
points(x,y,xlim=c(0,9), ylim=c(0,300),xlab="", ylab="", las=1, col='tomato',type='b', lwd=1.5, pch=20)
legend("topleft", c("WW","WD"), col=c("cornflowerblue","tomato"), pch=20, bty="n")
```

---

1. R\_Core\_Team: R: A Language and Environment for Statistical Computing. R 3.0.0 edition. Vienna, Austria: R Foundation for Statistical Computing; 2015.↩
2. Muggeo VM, Muggeo MVM: Package ‘segmented’. 2017.↩

LS0tDQp0aXRsZTogIlNpbGsgZ3Jvd3RoIGR5bmFtaWNzIGFuYWx5c2lzIg0KYXV0aG9yOiAiTmljb2xhcyBCcmljaGV0ICYgTGxvcmVuw6cgQ2FicmVyYS1Cb3NxdWV0Ig0Kb3V0cHV0OiBodG1sX25vdGVib29rDQotLS0NCg0KYGBge3Igc2V0dXAsIGluY2x1ZGU9RkFMU0V9DQprbml0cjo6b3B0c19jaHVuayRzZXQoZWNobyA9IFRSVUUpDQpgYGANCg0KDQojQW5hbHlzaXMgb2YgZWFyIGFuZCBzaWxrIGltYWdlcyB1c2luZyBJbGFzdGlrDQoNClBpeGVscyBjb3JyZXNwb25kaW5nIHRvIHNpbGtzIHdlcmUgZXh0cmFjdGVkIHVzaW5nIGEgcmFuZG9tIGZvcmVzdCBjbGFzc2lmaWNhdGlvbiBtZXRob2QgYmFzZWQgb24gY29sb3VyIChHYXVzc2lhbiBzbW9vdGhpbmcgb2YgNSBweCkgYW5kIHRleHR1cmUgKHN0cnVjdHVyZSB0ZW5zb3IgZWlnZW52YWx1ZXMgb2YgMS42IHB4KSBpbiBbKipJbGFzdGlrIHNvZnR3YXJlLCB2ZXJzaW9uIDEuMS43KipdKGh0dHA6Ly9pbGFzdGlrLm9yZy8pLg0KDQpUaGUgZm9sbG93aW5nIG1hdGVyaWFscyBhcmUgYXZhaWxhYmxlIGF0IFshW0RPSV0oaHR0cHM6Ly96ZW5vZG8ub3JnL2JhZGdlL0RPSS8xMC41MjgxL3plbm9kby4xMDAyMTczLnN2ZyldKGh0dHBzOi8vZG9pLm9yZy8xMC41MjgxL3plbm9kby4xMDAyMTczKSBmb3IgcmVwcm9kdWNpbmcgc2lsayBleHRyYWN0aW9uIGFuYWx5c2lzLg0KDQpBIGZvbGRlciBuYW1lZCBEQVRBU0VUIFNJTEtTIGNvbnRhaW5zOg0KDQoxLiBgVHJhaW5pbmcgZGF0YXNldGA6IGltYWdlIGRhdGFzZXQgb2YgY29udHJhc3RpbmcgZWFyIGltYWdlcyBpbnZvbHZpbmcgcGxhbnRzIG9mIGRpZmZlcmVudCBnZW5vdHlwZXMgYXQgZGlmZmVyZW50IGVhciBhbmQgc2lsayBkZXZlbG9wbWVudGFsIHN0YWdlcyANCg0KMi4gYFZhbGlkYXRpb24gZGF0YXNldGA6IGltYWdlIGRhdGFzZXQgb2YgMyB3ZWxsLXdhdGVyZWQgYW5kIDMgd2F0ZXItZGVmaWNpdCBwbGFudHMgb2YgdGhlIHNhbWUgZ2Vub3R5cGUNCg0KMy4gYElsYXN0aWsgcHJvamVjdGA6IHRoZSBmaWxlICoqc2lsa3NfY2xhc3NpZmljYXRpb25fbGlnaHQuaWxwKiogY29udGFpbnMgYWxsIGNvbG91ciBhbmQgdGV4dHVyZSBjbGFzc2lmaWNhdGlvbiBmZWF0dXJlcyB1c2luZyB0aGUgdHJhaW5pbmcgaW1hZ2UgZGF0YXNldA0KDQo0LiBgSWxhc3Rpa19vdXRwdXRgOiBpbWFnZSBkYXRhc2V0IG9mIElsYXN0aWsgb3V0cHV0IHJlc3VsdGluZyBpbWFnZXMgb2YgcGxhbnQgIzMxNiBwcmVzZW50IGluIHRoZSBgVmFsaWRhdGlvbiBkYXRhc2V0YA0KDQo1LiBgZGF0YV9zaWxrX2lsYXN0aWtfb3V0cHV0LmNzdmA6IGNzdiBmaWxlIGNvbnRhaW5pbmcgc2lsayBwaXhlbHMgZGF0YSBvZiA2IHBsYW50cw0KDQoNCg0KVGhlIHBpcGVsaW5lIGlzIGRlc2NyaWJlZCBpbiB0aGUgZm9sbG93aW5nIGZpZ3VyZToNCg0KDQohW10oLi9waXBlbGluZS5wbmcpDQoNCg0KI0V4dHJhY3Qgc2lsayBwaXhlbHMgZnJvbSBJbGFzdGlrIG91dHB1dHMNClRoaXMgZXhhbXBsZSBzaG93cyBob3cgdG8gZXh0cmFjdCBzaWxrIHBpeGVscyBmcm9tIElsYXN0aWsgb3V0cHV0cw0KDQpgYGB7ciwgbWVzc2FnZT1GQUxTRSwgd2FybmluZz1GQUxTRX0NCiNSZXF1aXJlZCBSIHBhY2thZ2VzDQppZiAoIXJlcXVpcmUoInBseXIiKSkgaW5zdGFsbC5wYWNrYWdlcygicGx5ciIpDQppZiAoIXJlcXVpcmUoIkVCSW1hZ2UiKSkgaW5zdGFsbC5wYWNrYWdlcygiRUJJbWFnZSIpDQpgYGANCg0KYGBge3IsIG1lc3NhZ2U9RkFMU0UsIHdhcm5pbmc9RkFMU0V9DQpwYXIobWZyb3c9YygxLDIpKQ0KY29saW1nID0gcmVhZEltYWdlKCIuL1RyYWluaW5nIGRhdGFzZXQvb3JpZ2luYWxzLzMxMF8yMDE3LTA2LTEwXzAzLTUxLTUyXzY4NTUucG5nIikNCmltZyA9IHJlYWRJbWFnZSgiLi9UcmFpbmluZyBkYXRhc2V0L2lsYXN0aWtfb3V0cHV0LzMxMF8yMDE3LTA2LTEwXzAzLTUxLTUyXzY4NTVfUHJvYmFiaWxpdGllcy5wbmciKQ0KDQpkaXNwbGF5KGNvbGltZywgbWV0aG9kPSdyYXN0ZXInKQ0KZGlzcGxheShpbWcsIG1ldGhvZD0ncmFzdGVyJykNCmltZ190aHJlc2ggPSBpbWcgPiAuNzUgI2FwcGx5IHRocmVzaG9sZA0KY29tcHV0ZUZlYXR1cmVzLnNoYXBlKGltZ190aHJlc2gpICNjb21wdXRlIGZlYXR1cmVzDQoNCmBgYA0KDQojRXhhbXBsZTogZXh0cmFjdCBkYXRhIGZyb20gdGVtcG9yYWwgc2VyaWVzDQoNClRoaXMgZXhlbXBsZSBzaG93cyBob3cgdG8gZXh0cmFjdCBzaWxrIHBpeGVscyBmcm9tIGEgc2V0IG9mIElsYXN0aWsgb3V0cHV0IGltYWdlcyBjb3JyZXNwb25kaW5nIHRvIHRoZSBwbGFudCAjMzE2IA0KDQpgYGB7ciwgbWVzc2FnZT1GQUxTRSwgd2FybmluZz1GQUxTRX0NCndkID0gIi4vSWxhc3Rpa19vdXRwdXQvIg0KZmlsZV9saXN0IDwtIGxpc3QuZmlsZXMod2QsIHBhdHRlcm4gPSAiLnBuZyIpDQoNCnRhYmxlX3Jlc3VsdHMgPC0gTlVMTA0KDQoNCmZvciAoZmlsZSBpbiBmaWxlX2xpc3Qpew0KIA0KaW1nID0gcmVhZEltYWdlKHBhc3RlKCIuL0lsYXN0aWtfb3V0cHV0LyIsZmlsZSxzZXA9IiIpKSAjcmVhZCBpbWFnZXMNCmltZ190aHJlc2ggPSBpbWcgPiAuNzUgI2FwcGx5IHRocmVzaG9sZA0KcmVzdWx0cyA8LSBjYmluZChmaWxlLGNvbXB1dGVGZWF0dXJlcy5zaGFwZShpbWdfdGhyZXNoKSkgI2NvbXB1dGUgZmVhdHVyZXMNCnRhYmxlX3Jlc3VsdHM9IGFzLmRhdGEuZnJhbWUocmJpbmQocmVzdWx0cyx0YWJsZV9yZXN1bHRzKSkNCg0KIH0NCg0KdGFibGVfcmVzdWx0cyRzLmFyZWE8LSBhcy5udW1lcmljKGFzLmNoYXJhY3Rlcih0YWJsZV9yZXN1bHRzJHMuYXJlYSkpDQp0YWJsZV9yZXN1bHRzIDwtIHRhYmxlX3Jlc3VsdHNbb3JkZXIodGFibGVfcmVzdWx0cyRmaWxlLCBkZWNyZWFzaW5nPVRSVUUpLF0NCmxpc3QgPC0gc3Ryc3BsaXQoYXMuY2hhcmFjdGVyKHRhYmxlX3Jlc3VsdHMkZmlsZSksICJfIikNCmRmIDwtIGxkcGx5KGxpc3QpDQpjb2xuYW1lcyhkZikgPC0gYygicGxhbnQiLCAiRGF5IiwiSGV1cmUiLCJ0YXNrIikNCiAgDQogIHRhYmxlX3Jlc3VsdHMkUGxhbnQgPC1kZiRwbGFudA0KICB0YWJsZV9yZXN1bHRzJERheSA8LSBkZiREYXkNCiAgdGFibGVfcmVzdWx0cyREYXkgPC0gYXMuRGF0ZSh0YWJsZV9yZXN1bHRzJERheSwgZm9ybWF0PSIlWS0lbS0lZCIpDQoNCiAgbGlzdCA8LSBzdHJzcGxpdChhcy5jaGFyYWN0ZXIoZGYkSGV1cmUpLCAiLnBuZyIpDQogIGRmIDwtIGxkcGx5KGxpc3QpDQogIGNvbG5hbWVzKGRmKSA8LSBjKCJIb3VyIikNCiAgdGFibGVfcmVzdWx0cyRIb3VyIDwtIGdzdWIoIi0iLCAiOiIsIGRmJEhvdXIpDQoNCiAgdGFibGVfcmVzdWx0cyREYXRlIDwtYXMuY2hhcmFjdGVyKHBhc3RlKHRhYmxlX3Jlc3VsdHMkRGF5LCIiLHRhYmxlX3Jlc3VsdHMkSG91cikpDQogIHRhYmxlX3Jlc3VsdHMkRGF0ZSA8LWFzLlBPU0lYY3QodGFibGVfcmVzdWx0cyREYXRlLCBmb3JtYXQ9IiVZLSVtLSVkICVIOiVNOiVTIikNCiAgDQogIHBsb3QodGFibGVfcmVzdWx0cyREYXRlLCB0YWJsZV9yZXN1bHRzJHMuYXJlYS8xMDAwLCB0eXBlPSdsJywgbHdkPTIsIGNvbD0yLCB5bGFiPSJTaWxrIHBpeGVscyAoMTBeMykiLCB4bGFiPSJEYXRlIiwgbGFzPTEpDQoNCmBgYA0KDQoNCiNHZXQgZGF0YSBvdXRwdXQgZnJvbSBJbGFzdGlrDQpUaGlzIGV4ZW1wbGUgaXMgYmFzZWQgb24gYSBjc3YgZmlsZSB0aGF0IGNvbnRhaW5zIHNpbGsgcGl4ZWxzIGZyb20gNiBwbGFudHMgY29ycmVzcG9uZGluZyB0byB0aGUgc2FtZSBnZW5vdHlwZQ0KDQpgYGB7ciwgbWVzc2FnZT1GQUxTRSwgd2FybmluZz1GQUxTRX0NCg0KI0xvYWQgY3N2IGZpbGUNCmRhdGFfb3V0cHV0IDwtIHJlYWQuY3N2KCIuL2RhdGFfc2lsa19pbGFzdGlrX291dHB1dC5jc3YiKQ0Ka25pdHI6OmthYmxlKGhlYWQoZGF0YV9vdXRwdXQpKQ0KYGBgDQoNCiNDb21wdXRlIHNsb3BlcyB1c2luZyBzZWdtZW50ZWQgcGFja2FnZQ0KDQpJbiB0aGlzIGV4ZW1wbGUgdGltZSBjb3Vyc2VzIG9mIHBpeGVscyBjb3JyZXNwb25kaW5nIHRvIHNpbGsgYnVuZGxlcyBhcmUgaW5kaXZpZHVhbGx5IGZpdHRlZCBmb3IgZWFjaCBwbGFudCB1c2luZyB0aGUgUiBwYWNrYWdlIF5bUl9Db3JlX1RlYW06IFI6IEEgTGFuZ3VhZ2UgYW5kIEVudmlyb25tZW50IGZvciBTdGF0aXN0aWNhbCBDb21wdXRpbmcuIFIgMy4wLjAgZWRpdGlvbi4gVmllbm5hLCBBdXN0cmlhOiBSIEZvdW5kYXRpb24gZm9yIFN0YXRpc3RpY2FsIENvbXB1dGluZzsgMjAxNS5dIOKAmHNlZ21lbnRlZOKAmSBeW011Z2dlbyBWTSwgTXVnZ2VvIE1WTTogUGFja2FnZSDigJhzZWdtZW50ZWTigJkuIDIwMTcuXSwgYW5kIHRoZSBtYXhpbXVtIHJhdGVzIG9mIHNpbGsgZ3Jvd3RoIGFuZCBkdXJhdGlvbiBvZiBzaWxrIGdyb3d0aCBhcmUgZXh0cmFjdGVkLg0KDQoNCg0KYGBge3IsIG1lc3NhZ2U9RkFMU0UsIHdhcm5pbmc9RkFMU0V9DQojUmVxdWlyZWQgUiBwYWNrYWdlcw0KaWYgKCFyZXF1aXJlKCJkcGx5ciIpKSBpbnN0YWxsLnBhY2thZ2VzKCJkcGx5ciIpDQppZiAoIXJlcXVpcmUoInNlZ21lbnRlZCIpKSBpbnN0YWxsLnBhY2thZ2VzKCJzZWdtZW50ZWQiKQ0KaWYgKCFyZXF1aXJlKCJsdWJyaWRhdGUiKSkgaW5zdGFsbC5wYWNrYWdlcygibHVicmlkYXRlIikNCmBgYA0KDQoNCg0KYGBge3IsIG1lc3NhZ2U9RkFMU0UsIHdhcm5pbmc9RkFMU0V9DQojdGFibGUgZm9yIHJlc3VsdHMNCnNlZ21lbnRlZG1ldGhvZHRhYmxlPU5VTEwNCg0KI0ZvciBpIGxvb3ANCmZvciAoaSBpbiBsZXZlbHMoZmFjdG9yKGRhdGFfb3V0cHV0JFBsYW50KSkpew0KICANCiAgc2VnbWVudGVkcGxhbnQgPSBkYXRhX291dHB1dFtkYXRhX291dHB1dCRQbGFudD09aSxdDQogIA0KICB4IDwtc2VnbWVudGVkcGxhbnQkZGFzDQogIHkgPC1zZWdtZW50ZWRwbGFudCRwaXhlbHNfbnVtYmVyDQogIA0KICBwIDwtIE5VTEwNCiAgICB0cnkocCA8LSBsbSh5fngpLHNpbGVudD1UUlVFKQ0KICBsIDwtIE5VTEwNCiAgICB0cnkobCA8LSBzZWdtZW50ZWQocCwgc2VnLlogPX54LCBwc2kgPSBsaXN0KHg9Yyg0KSkpLHNpbGVudD1UKQ0KICANCiAgU0VSIDwtIGlmZWxzZShpcy5udWxsKGwpPT1ULE5BLHNsb3BlKGwpJHhbMV0pICNzaWxrIGV4cGFuc2lvbiByYXRlDQogIER1cmF0aW9uIDwtaWZlbHNlKGlzLm51bGwobCk9PVQsTkEsc3VtbWFyeShsKSRwc2lbMl0pICNzaWxrIGdyb3d0aCBkdXJhdGlvbiAoYnJlYWtwb2ludCkNCiAgbmV3bGluZSA8LSBkYXRhLmZyYW1lKElEPSBpLCBTRVIgPSBhcy5udW1lcmljKGFzLmNoYXJhY3RlcihTRVIpKSwgRHVyYXRpb249YXMubnVtZXJpYyhhcy5jaGFyYWN0ZXIoRHVyYXRpb24pKSkNCiAgc2VnbWVudGVkbWV0aG9kdGFibGUgPC1yYmluZChzZWdtZW50ZWRtZXRob2R0YWJsZSwgbmV3bGluZSkNCiAgDQp9DQpzZWdtZW50ZWRtZXRob2R0YWJsZSRTY2VuYXJpbyA8LSBkYXRhX291dHB1dCRTY2VuYXJpb1ttYXRjaChzZWdtZW50ZWRtZXRob2R0YWJsZSRJRCxkYXRhX291dHB1dCRwbGFudCldDQoNCmBgYA0KDQojUmVzdWx0cw0KYGBge3IsIGZpZy5oZWlnaHQ9NSwgZmlnLndpZHRoPTEwLCBtZXNzYWdlPUZBTFNFLCB3YXJuaW5nPUZBTFNFfQ0KcGFyKG1mcm93PWMoMSwyKSkNCmJveHBsb3Qoc2VnbWVudGVkbWV0aG9kdGFibGUkU0VSfnNlZ21lbnRlZG1ldGhvZHRhYmxlJFNjZW5hcmlvLCBjb2w9YygidG9tYXRvIiwiY29ybmZsb3dlcmJsdWUiKSwgbWFpbj0gIlNpbGsgZ3Jvd3RoIHJhdGUgKHBpeGVsIGQtMSkiLCBsYXM9MSkNCmJveHBsb3Qoc2VnbWVudGVkbWV0aG9kdGFibGUkRHVyYXRpb25+c2VnbWVudGVkbWV0aG9kdGFibGUkU2NlbmFyaW8sIGNvbD1jKCJ0b21hdG8iLCJjb3JuZmxvd2VyYmx1ZSIpLCBtYWluPSAiU2lsayBncm93dGggZHVyYXRpb24gKGQpIiwgbGFzPTEpDQpgYGANCg0KI01lYW4gc2lsayBleHBhbnNpb24gcmF0ZSBhbmQgc2lsayBncm93dGggZHVyYXRpb24NCmBgYHtyLCBlY2hvPUZBTFNFLCBtZXNzYWdlPUZBTFNFLCB3YXJuaW5nPUZBTFNFLCByZXN1bHRzPSdhc2lzJ30NCmluZm9leHAgPC1hZ2dyZWdhdGUoc2VnbWVudGVkbWV0aG9kdGFibGVbYygiU0VSIiwiRHVyYXRpb24iKV0sIGJ5PXNlZ21lbnRlZG1ldGhvZHRhYmxlWyJTY2VuYXJpbyJdLCBGVU49bWVhbiwgbmEucm09VCkNCmtuaXRyOjprYWJsZShoZWFkKGluZm9leHApKQ0KYGBgDQoNCg0KI1NpbGsgZ3Jvd3RoIGR5bmFtaWNzDQoNCmBgYHtyLCBtZXNzYWdlPUZBTFNFLCB3YXJuaW5nPUZBTFNFfQ0KcGFyKGJnPU5BKQ0KdGVzdCA9IGFnZ3JlZ2F0ZShkYXRhX291dHB1dFtjKCAicGl4ZWxzX251bWJlciIpXSwgYnk9ZGF0YV9vdXRwdXRbYygiZmxvIiwiU2NlbmFyaW8iKV0sIEZVTj1tZWFuLCBuYS5ybT1UKQ0KdGVzdCA9IHRlc3RbdGVzdCRmbG8+LTMsXSAjbGltaXQgdG8gMyBkYXlzIGJlZm9yZSBmbG93ZXJpbmcNCnRlc3RzZCA9IGFnZ3JlZ2F0ZShkYXRhX291dHB1dFtjKCJwaXhlbHNfbnVtYmVyIildLCBieT1kYXRhX291dHB1dFtjKCJmbG8iLCJTY2VuYXJpbyIpXSwgRlVOPXNkLCBuYS5ybT1UKQ0KdGVzdHNkID0gdGVzdHNkW3Rlc3RzZCRmbG8+LTMsXSAjbGltaXQgdG8gMyBkYXlzIGJlZm9yZSBmbG93ZXJpbmcNCg0KI1dXIHBsYW50cw0Ka2sgPSB0ZXN0W3Rlc3QkU2NlbmFyaW89PSJXVyIsXQ0Ka2skY29sb3IgPSBpZmVsc2UgKGtrJFNjZW5hcmlvPT0iV1ciLCAiY29ybmZsb3dlcmJsdWUiLCAidG9tYXRvIikNCg0KeD0ga2skZmxvDQp5PSBrayRwaXhlbHNfbnVtYmVyLzEwMDANCnkuRXJyID0gbmEub21pdCh0ZXN0c2RbdGVzdHNkJFNjZW5hcmlvPT0iV1ciLF0kcGl4ZWxzX251bWJlci8xMDAwKS8gc3FydChucm93KHRlc3RzZFt0ZXN0c2QkU2NlbmFyaW89PSJXVyIsXSktMSkNCnkuVXAgPSB5ICsgeS5FcnINCnkuRG4gPSB5IC0geS5FcnINCg0KcGxvdCh4LHkseGxpbT1jKDAsOSksIHlsaW09YygwLDMwMCkseGxhYj0iRGF5cyBhZnRlciBmbG93ZXJpbmciLCB5bGFiPSJTaWxrIHBpeGVscyAoMTBeMykiLCBsYXM9MSwgY29sPSdjb3JuZmxvd2VyYmx1ZScsdHlwZT0nYicsIGx3ZD0xLjUsIHBjaD0yMCkNCmdyaWQoKQ0KcG9seWdvbihjKHgscmV2KHgpKSxjKHkuVXAscmV2KHkuRG4pKSxjb2w9ImdyZXk5MCIsYm9yZGVyPSJ3aGl0ZSIpDQpwb2ludHMoeCx5LHhsaW09YygwLDkpLCB5bGltPWMoMCwzMDApLHhsYWI9IiIsIHlsYWI9IiIsIGxhcz0xLCBjb2w9J2Nvcm5mbG93ZXJibHVlJyx0eXBlPSdiJywgbHdkPTEuNSwgcGNoPTIwKQ0KDQojV0QgcGxhbnRzDQprayA9IHRlc3RbdGVzdCRTY2VuYXJpbz09IldEIixdDQprayRjb2xvciA9IGlmZWxzZSAoa2skU2NlbmFyaW89PSJXVyIsICJjb3JuZmxvd2VyYmx1ZSIsICJ0b21hdG8iKQ0KDQp4PSBrayRmbG8NCnk9IGtrJHBpeGVsc19udW1iZXIvMTAwMA0KeS5FcnIgPSBuYS5vbWl0KHRlc3RzZFt0ZXN0c2QkU2NlbmFyaW89PSJXRCIsXSRwaXhlbHNfbnVtYmVyLzEwMDApLyBzcXJ0KG5yb3codGVzdHNkW3Rlc3RzZCRTY2VuYXJpbz09IldEIixdKS0xKQ0KeS5VcCA9IHkgKyB5LkVycg0KeS5EbiA9IHkgLSB5LkVycg0KDQpwb2ludHMoeCx5LHhsaW09YygwLDkpLCB5bGltPWMoMCwzMDApLHhsYWI9IiIsIHlsYWI9IiIsIGxhcz0xLCBjb2w9J2Nvcm5mbG93ZXJibHVlJyx0eXBlPSdiJywgbHdkPTEuNSwgcGNoPTIwKQ0KcG9seWdvbihjKHgscmV2KHgpKSxjKHkuVXAscmV2KHkuRG4pKSxjb2w9ImdyZXk5MCIsYm9yZGVyPSJ3aGl0ZSIsIGFkZD1UKQ0KcG9pbnRzKHgseSx4bGltPWMoMCw5KSwgeWxpbT1jKDAsMzAwKSx4bGFiPSIiLCB5bGFiPSIiLCBsYXM9MSwgY29sPSd0b21hdG8nLHR5cGU9J2InLCBsd2Q9MS41LCBwY2g9MjApDQpsZWdlbmQoInRvcGxlZnQiLCBjKCJXVyIsIldEIiksIGNvbD1jKCJjb3JuZmxvd2VyYmx1ZSIsInRvbWF0byIpLCBwY2g9MjAsIGJ0eT0ibiIpDQpgYGANCg0K
