# Supplementary material for: A robot-assisted imaging pipeline for tracking the growths of maize ear and silks in a high-throughput phenotyping platform
Source: Plant Methods. 2017 Nov 8;13:96. doi: 10.1186/s13007-017-0246-7 (PMC5688816; doi:10.1186/s13007-017-0246-7)
Supplement: Supplementary file 4 — Additional file 4. R HTML notebook allowing reproducing Fig. 5. [file 13007_2017_246_MOESM4_ESM.html]

Whole plant segmentation


Code 

- Show All Code
- Hide All Code
- Download Rmd

# Whole plant segmentation

#### *Nicolas Brichet & Llorenç Cabrera-Bosquet*

This script allows to reproduce Figure 5 that shows plant representations at different development stages (one to eight weeks after sowing) for (a) top and (b) side images, and time courses of the number of pixels corresponding to plants extracted from side and top views (c)

The following materials are available at

A folder named `Maize whole plant image dataset` contains:

1. `Image dataset`: image dataset of side and top RGB images of a single plant that can be used in the segmentation pipeline (https://github.com/openalea/eartrack)
2. `Segmented image dataset`: output images of the segmentation pipeline
3. `Image analysis features`: csv file containing all image analysis features from the image dataset provided above

# Figure 5a,b

Plant representations at different developmental stages (one to eight weeks after sowing) for (a) top and (b) side images


```
#Required R packages
if (!require("EBImage")) install.packages("EBImage")
```


```
par(mfrow=c(2,5))
#top images
topimage= list.files("./FIG5/top/")
for(image in topimage){
  imageiday = readImage(paste("./FIG5/top/", image,sep=""))
  display(imageiday, method='raster')
}
#side images
sideimage= list.files("./FIG5/side/")
for(image in sideimage){
  imageiday = readImage(paste("./FIG5/side/", image,sep=""))
  display(imageiday, method='raster')
}
```

# Get output data from segmentation pipeline

This exemple is based on a csv file that contains image analysis features from one single plant


```
#Load csv file
data_output <- read.csv("./segmentationdata.csv")
knitr::kable(head(data_output))
```


| datetime | viewtypeid | angle | convex\_hull\_perimeter | width | convex\_hull\_area | object\_sum\_area | height\_over\_pot | height\_under\_pot | height | Sowing | Day | DAS |
| --- | --- | --- | --- | --- | --- | --- | --- | --- | --- | --- | --- | --- |
| 14/04/2017 01:06 | 1 | 0 | 219.1609 | 71 | 2231.5 | 1577 | NA | NA | 71 | 96 | 14/04/2017 | 8 |
| 14/04/2017 01:06 | 2 | 0 | 303.7828 | 50 | 3066.0 | 1713 | 138 | 0 | 137 | 96 | 14/04/2017 | 8 |
| 14/04/2017 01:06 | 2 | 330 | 278.4993 | 31 | 1549.0 | 1171 | 134 | 0 | 133 | 96 | 14/04/2017 | 8 |
| 14/04/2017 01:06 | 2 | 300 | 273.5317 | 32 | 2371.5 | 1382 | 131 | 0 | 130 | 96 | 14/04/2017 | 8 |
| 14/04/2017 01:06 | 2 | 270 | 295.2435 | 55 | 3674.5 | 1515 | 132 | 0 | 131 | 96 | 14/04/2017 | 8 |
| 14/04/2017 01:06 | 2 | 240 | 313.4547 | 70 | 4645.5 | 1663 | 133 | 0 | 132 | 96 | 14/04/2017 | 8 |

# Figure 5c

Time courses of the number of pixels corresponding to plants extracted from side and top views.


```
par(bg=NA)
plot(object_sum_area/10^3~DAS, type='b', data=data_output[data_output$viewtypeid==1,], las=1, col=2, pch=1, ylab='Pixel number (10^3)', xlab="Days after sowing")
points(object_sum_area/10^3~DAS, type='b', data=data_output[data_output$angle==90,], col=2, pch=0, ylab='', las=1)
```


```
legend("topleft", c("Top view","Side 90° view"), pch=c(1,0), col=c(2,2), bty='n')
```

LS0tDQp0aXRsZTogIldob2xlIHBsYW50IHNlZ21lbnRhdGlvbiINCmF1dGhvcjogIk5pY29sYXMgQnJpY2hldCAmIExsb3JlbsOnIENhYnJlcmEtQm9zcXVldCINCm91dHB1dDogaHRtbF9ub3RlYm9vaw0KLS0tDQoNCg0KVGhpcyBzY3JpcHQgYWxsb3dzIHRvIHJlcHJvZHVjZSBGaWd1cmUgNSB0aGF0IHNob3dzIHBsYW50IHJlcHJlc2VudGF0aW9ucyBhdCBkaWZmZXJlbnQgZGV2ZWxvcG1lbnQgc3RhZ2VzIChvbmUgdG8gZWlnaHQgd2Vla3MgYWZ0ZXIgc293aW5nKSBmb3IgKGEpIHRvcCAgYW5kIChiKSBzaWRlIGltYWdlcywgYW5kIHRpbWUgY291cnNlcyBvZiB0aGUgbnVtYmVyIG9mIHBpeGVscyBjb3JyZXNwb25kaW5nIHRvIHBsYW50cyBleHRyYWN0ZWQgZnJvbSBzaWRlIGFuZCB0b3Agdmlld3MgKGMpIA0KDQpUaGUgZm9sbG93aW5nIG1hdGVyaWFscyBhcmUgYXZhaWxhYmxlIGF0IFshW0RPSV0oaHR0cHM6Ly96ZW5vZG8ub3JnL2JhZGdlL0RPSS8xMC41MjgxL3plbm9kby4xMDAyNjc1LnN2ZyldKGh0dHBzOi8vZG9pLm9yZy8xMC41MjgxL3plbm9kby4xMDAyNjc1KQ0KDQpBIGZvbGRlciBuYW1lZCBgTWFpemUgd2hvbGUgcGxhbnQgaW1hZ2UgZGF0YXNldGAgY29udGFpbnM6DQoNCjEuIGBJbWFnZSBkYXRhc2V0YDogaW1hZ2UgZGF0YXNldCBvZiBzaWRlIGFuZCB0b3AgUkdCIGltYWdlcyBvZiBhIHNpbmdsZSBwbGFudCB0aGF0IGNhbiBiZSB1c2VkIGluIHRoZSBzZWdtZW50YXRpb24gcGlwZWxpbmUgKGh0dHBzOi8vZ2l0aHViLmNvbS9vcGVuYWxlYS9lYXJ0cmFjaykNCg0KMi4gYFNlZ21lbnRlZCBpbWFnZSBkYXRhc2V0YDogb3V0cHV0IGltYWdlcyBvZiB0aGUgc2VnbWVudGF0aW9uIHBpcGVsaW5lDQoNCjMuIGBJbWFnZSBhbmFseXNpcyBmZWF0dXJlc2A6IGNzdiBmaWxlIGNvbnRhaW5pbmcgYWxsIGltYWdlIGFuYWx5c2lzIGZlYXR1cmVzIGZyb20gdGhlIGltYWdlIGRhdGFzZXQgcHJvdmlkZWQgYWJvdmUNCg0KDQoNCiNGaWd1cmUgNWEsYg0KUGxhbnQgcmVwcmVzZW50YXRpb25zIGF0IGRpZmZlcmVudCBkZXZlbG9wbWVudGFsIHN0YWdlcyAob25lIHRvIGVpZ2h0IHdlZWtzIGFmdGVyIHNvd2luZykgZm9yIChhKSB0b3AgYW5kIChiKSBzaWRlIGltYWdlcw0KDQpgYGB7ciwgbWVzc2FnZT1GQUxTRSwgd2FybmluZz1GQUxTRX0NCiNSZXF1aXJlZCBSIHBhY2thZ2VzDQppZiAoIXJlcXVpcmUoIkVCSW1hZ2UiKSkgaW5zdGFsbC5wYWNrYWdlcygiRUJJbWFnZSIpDQpgYGANCg0KYGBge3IsIG1lc3NhZ2U9RkFMU0UsIHdhcm5pbmc9RkFMU0V9DQpwYXIobWZyb3c9YygyLDUpKQ0KDQojdG9wIGltYWdlcw0KdG9waW1hZ2U9IGxpc3QuZmlsZXMoIi4vRklHNS90b3AvIikNCg0KZm9yKGltYWdlIGluIHRvcGltYWdlKXsNCiAgaW1hZ2VpZGF5ID0gcmVhZEltYWdlKHBhc3RlKCIuL0ZJRzUvdG9wLyIsIGltYWdlLHNlcD0iIikpDQogIGRpc3BsYXkoaW1hZ2VpZGF5LCBtZXRob2Q9J3Jhc3RlcicpDQp9DQoNCiNzaWRlIGltYWdlcw0Kc2lkZWltYWdlPSBsaXN0LmZpbGVzKCIuL0ZJRzUvc2lkZS8iKQ0KDQpmb3IoaW1hZ2UgaW4gc2lkZWltYWdlKXsNCiAgaW1hZ2VpZGF5ID0gcmVhZEltYWdlKHBhc3RlKCIuL0ZJRzUvc2lkZS8iLCBpbWFnZSxzZXA9IiIpKQ0KICBkaXNwbGF5KGltYWdlaWRheSwgbWV0aG9kPSdyYXN0ZXInKQ0KfQ0KDQpgYGANCg0KI0dldCBvdXRwdXQgZGF0YSBmcm9tIHNlZ21lbnRhdGlvbiBwaXBlbGluZQ0KVGhpcyBleGVtcGxlIGlzIGJhc2VkIG9uIGEgY3N2IGZpbGUgdGhhdCBjb250YWlucyBpbWFnZSBhbmFseXNpcyBmZWF0dXJlcyBmcm9tIG9uZSBzaW5nbGUgcGxhbnQNCg0KYGBge3IsIG1lc3NhZ2U9RkFMU0UsIHdhcm5pbmc9RkFMU0V9DQojTG9hZCBjc3YgZmlsZQ0KZGF0YV9vdXRwdXQgPC0gcmVhZC5jc3YoIi4vc2VnbWVudGF0aW9uZGF0YS5jc3YiKQ0Ka25pdHI6OmthYmxlKGhlYWQoZGF0YV9vdXRwdXQpKQ0KYGBgDQoNCiNGaWd1cmUgNWMNClRpbWUgY291cnNlcyBvZiB0aGUgbnVtYmVyIG9mIHBpeGVscyBjb3JyZXNwb25kaW5nIHRvIHBsYW50cyBleHRyYWN0ZWQgZnJvbSBzaWRlIGFuZCB0b3Agdmlld3MuDQoNCmBgYHtyLCBtZXNzYWdlPUZBTFNFLCB3YXJuaW5nPUZBTFNFfQ0KcGFyKGJnPU5BKQ0KcGxvdChvYmplY3Rfc3VtX2FyZWEvMTBeM35EQVMsIHR5cGU9J2InLCBkYXRhPWRhdGFfb3V0cHV0W2RhdGFfb3V0cHV0JHZpZXd0eXBlaWQ9PTEsXSwgbGFzPTEsIGNvbD0yLCBwY2g9MSwgeWxhYj0nUGl4ZWwgbnVtYmVyICgxMF4zKScsIHhsYWI9IkRheXMgYWZ0ZXIgc293aW5nIikNCg0KcG9pbnRzKG9iamVjdF9zdW1fYXJlYS8xMF4zfkRBUywgdHlwZT0nYicsIGRhdGE9ZGF0YV9vdXRwdXRbZGF0YV9vdXRwdXQkYW5nbGU9PTkwLF0sIGNvbD0yLCBwY2g9MCwgeWxhYj0nJywgbGFzPTEpDQpsZWdlbmQoInRvcGxlZnQiLCBjKCJUb3AgdmlldyIsIlNpZGUgOTDCsCB2aWV3IiksIHBjaD1jKDEsMCksIGNvbD1jKDIsMiksIGJ0eT0nbicpDQoNCmBgYA0K
