# Supplementary material for: A robot-assisted imaging pipeline for tracking the growths of maize ear and silks in a high-throughput phenotyping platform
Source: Plant Methods. 2017 Nov 8;13:96. doi: 10.1186/s13007-017-0246-7 (PMC5688816; doi:10.1186/s13007-017-0246-7)
Supplement: Supplementary file 9 — Additional file 9. Silk growth dynamics of the 60 studied maize lines grown under well-watered (WW) and water deficit (WD) conditions. Points, mean of three replicates, shaded areas, standard error. [file 13007_2017_246_MOESM9_ESM.pdf]

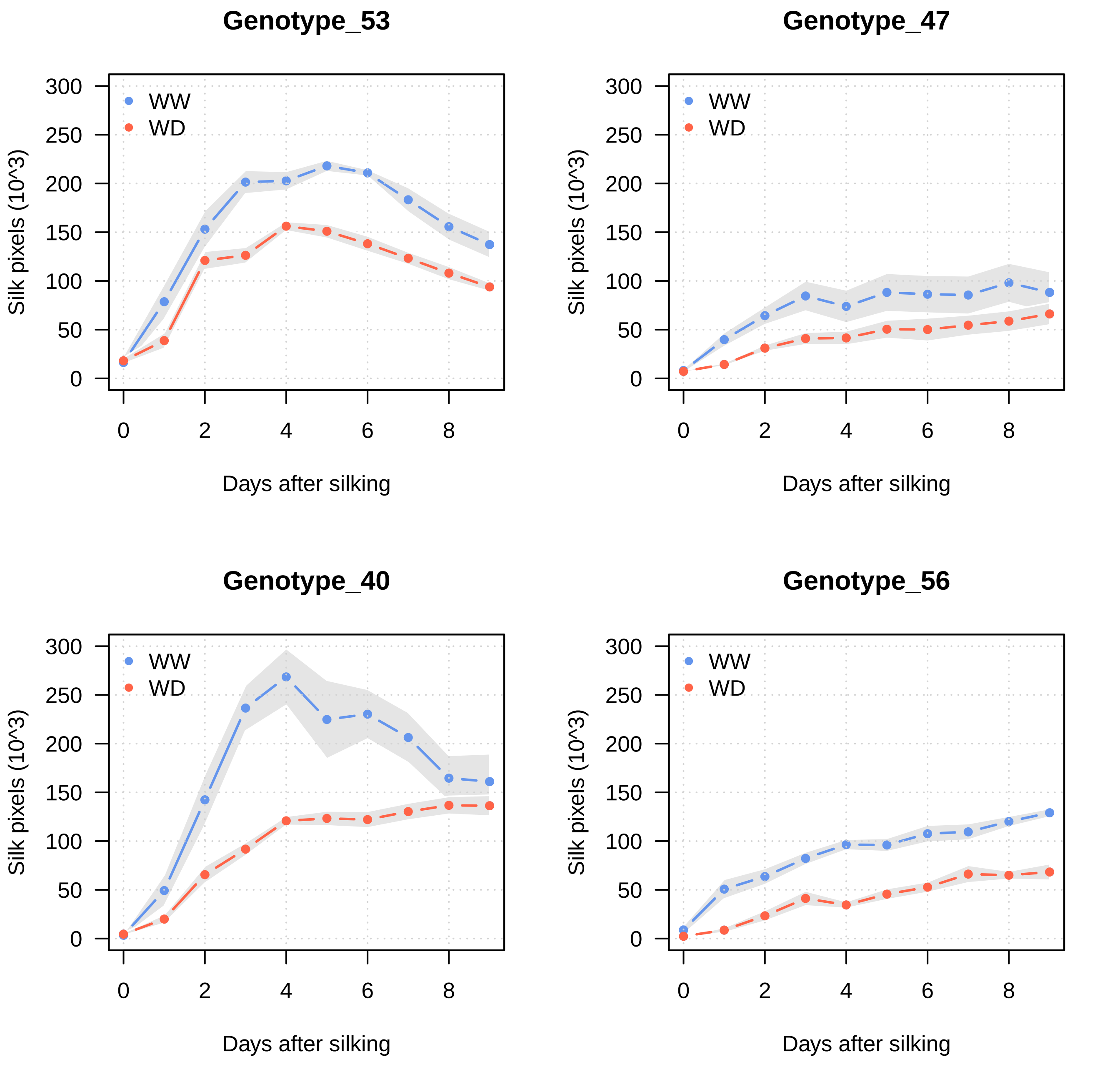

Genotype_53
Genotype_47
Genotype_40
Genotype_56
WW
WD
Silk pixels (10^3)
Days after silking
0
50
100
150
200
250
300
0
2
4
6
8

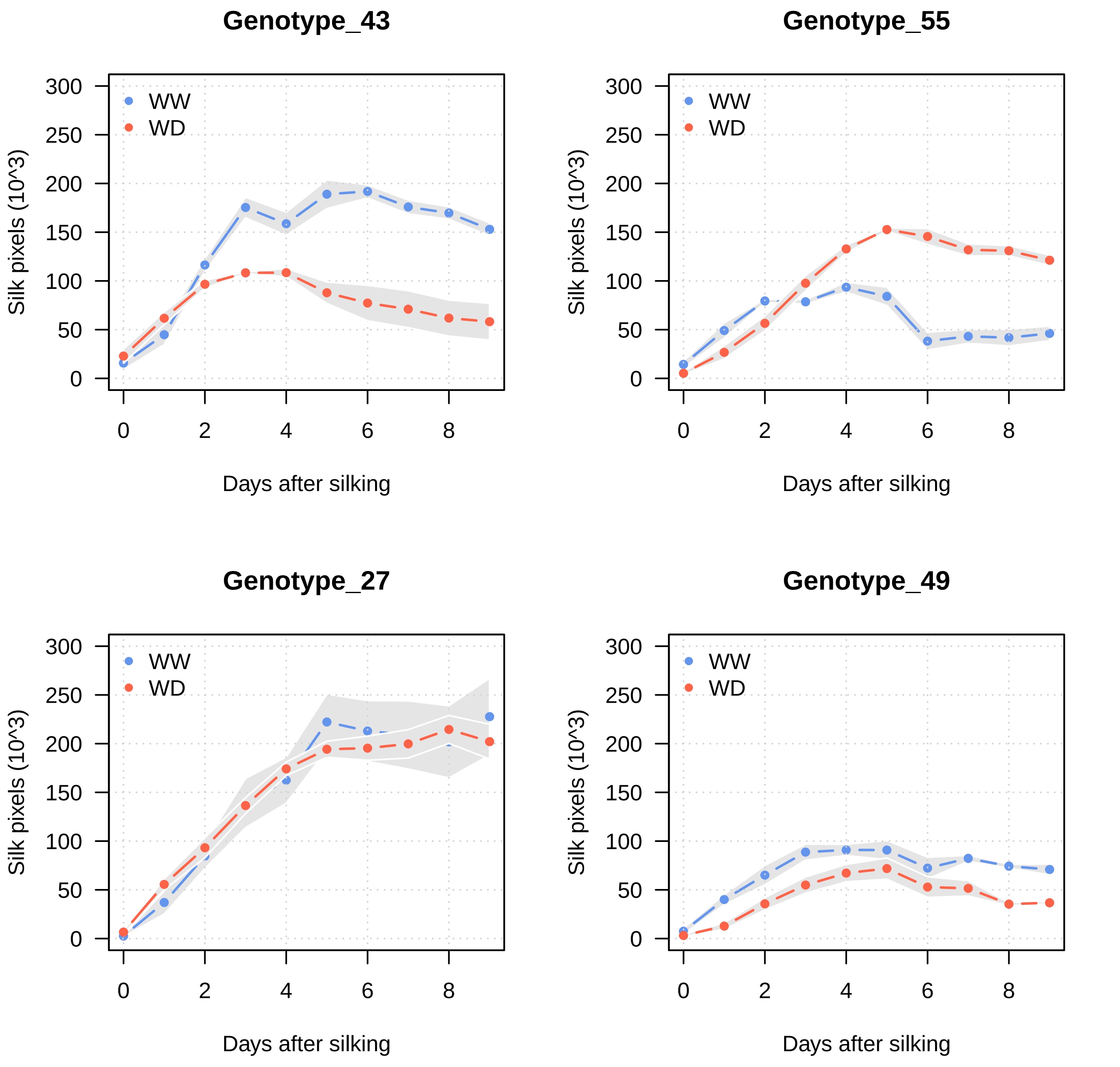
Genotype_43
Genotype_55
Genotype_27
Genotype_49
WW
WD
Silk pixels (10^3)
Days after silking
0
50
100
150
200
250
300
0
2
4
6
8

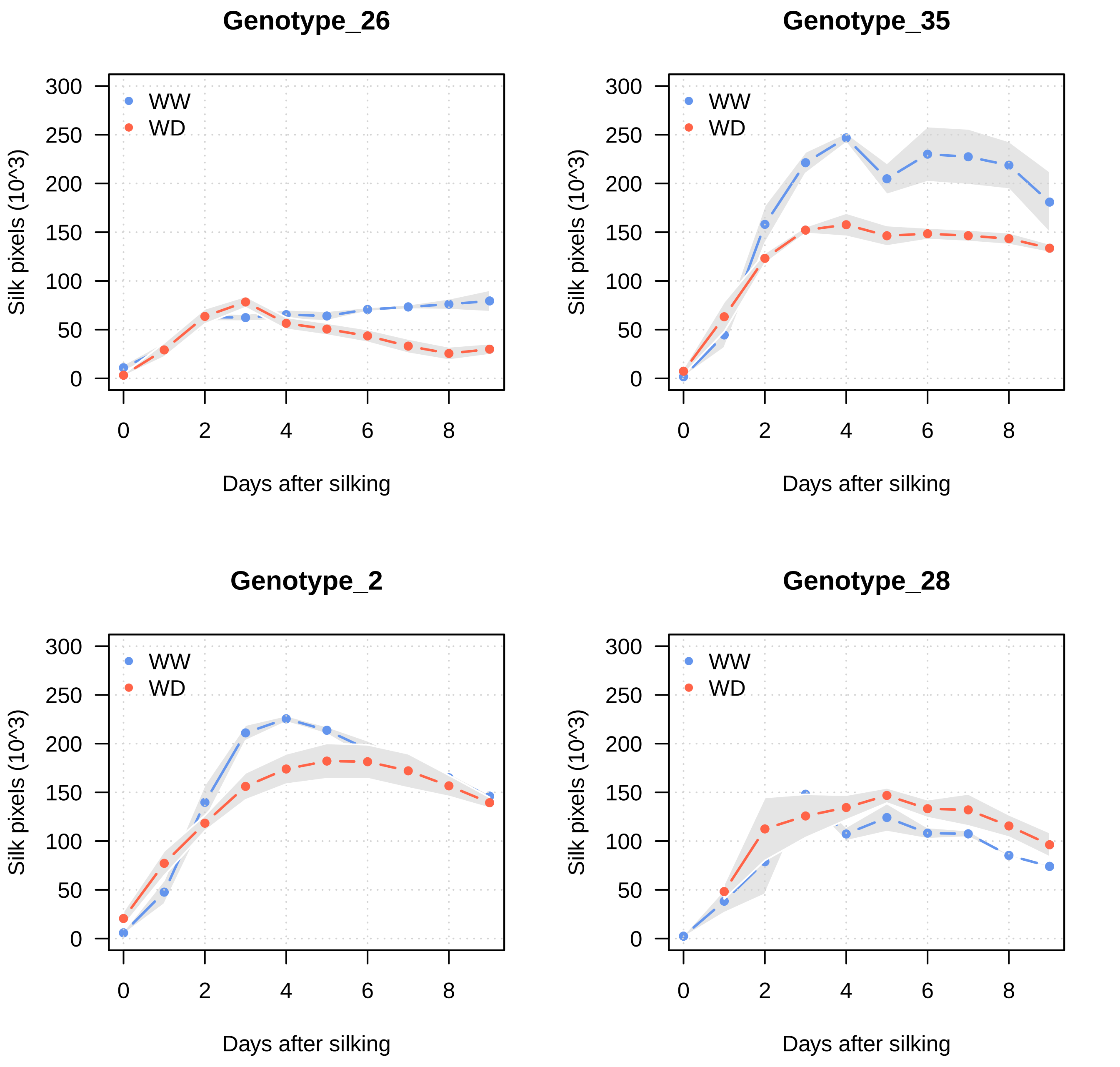
Genotype_26
Genotype_35
Genotype_2
Genotype_28
WW
WD
Silk pixels (10^3)
Days after silking
0
50
100
150
200
250
300
0
2
4
6
8

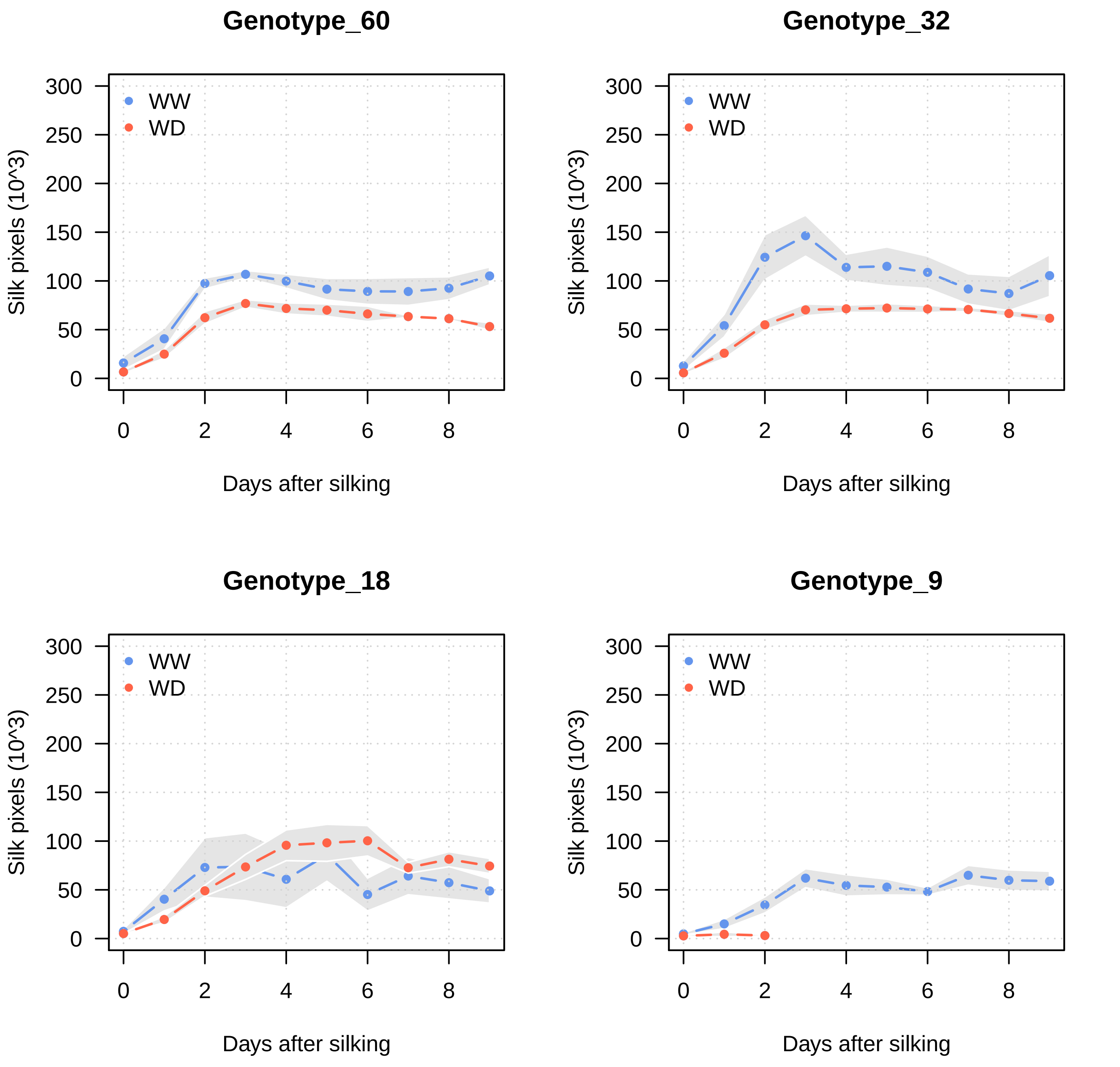
Genotype_60
Genotype_32
Genotype_18
Genotype_9
WW
WD
Silk pixels (10^3)
Days after silking
0
50
100
150
200
250
300
0
2
4
6
8

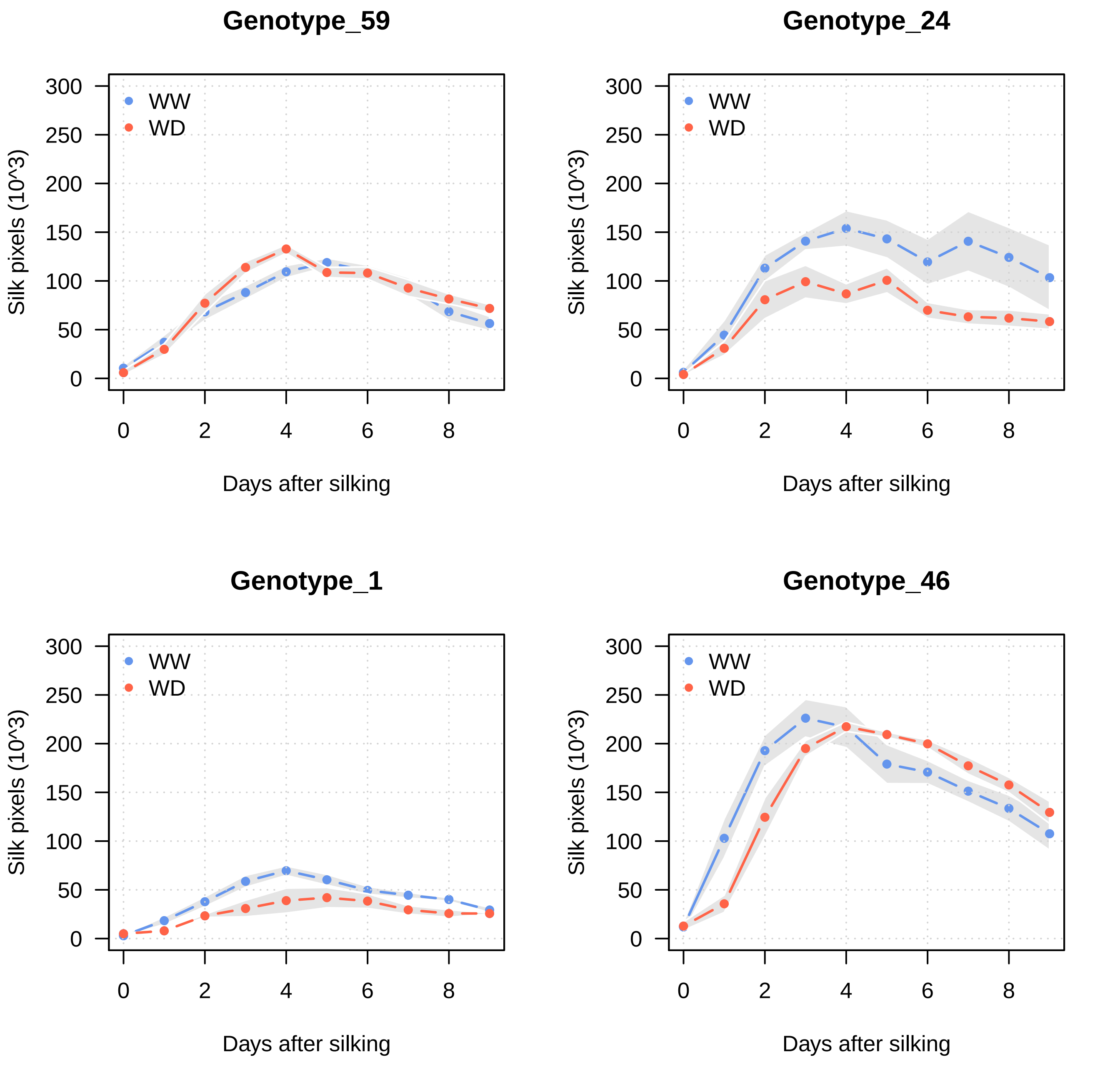
Genotype_59
Genotype_24
Genotype_1
Genotype_46
WW
WD
Silk pixels (10^3)
Days after silking
0
50
100
150
200
250
300
0
2
4
6
8

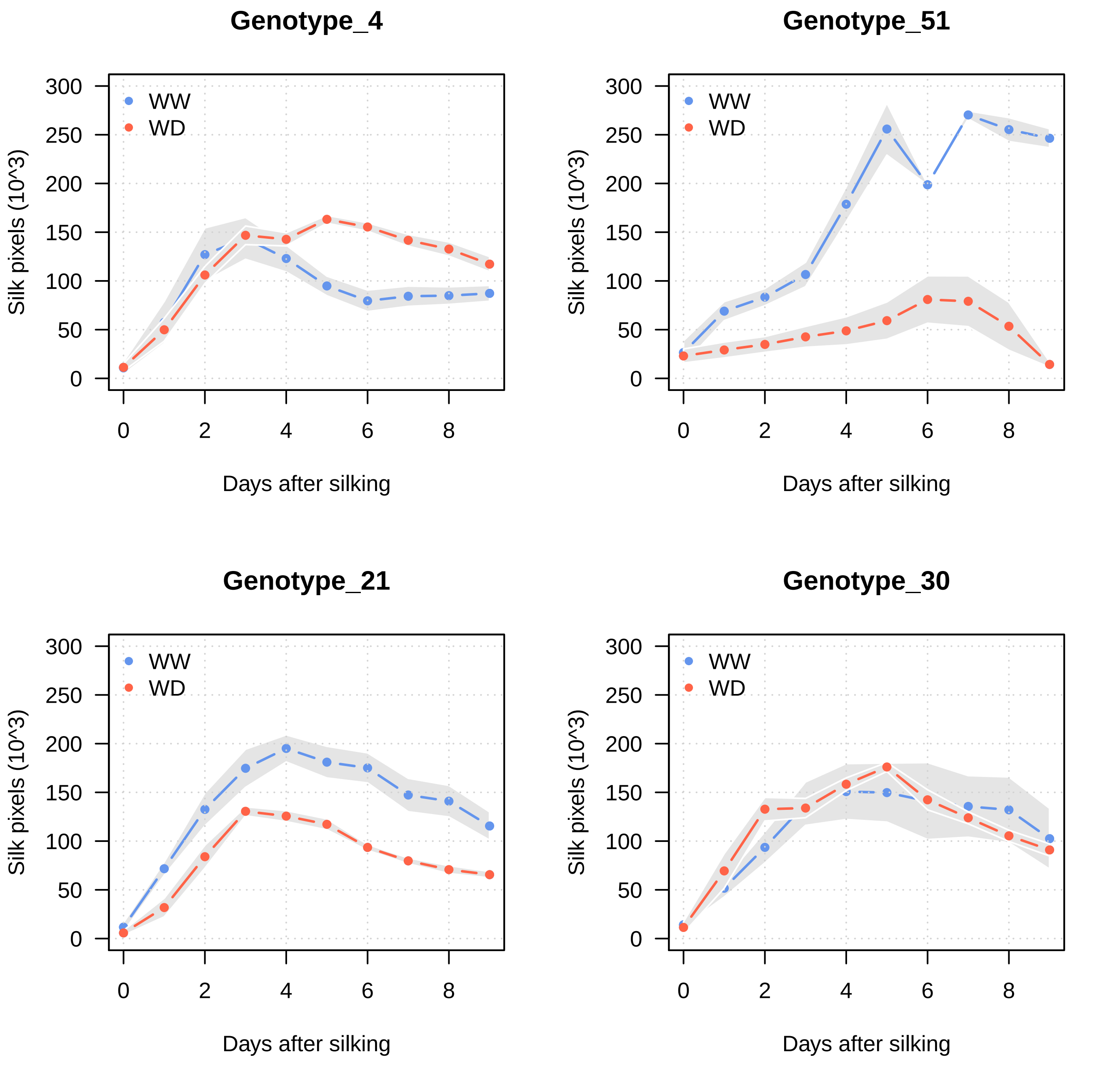
Genotype_4
Genotype_51
Genotype_21
Genotype_30
WW
WD
Silk pixels (10^3)
Days after silking
0
50
100
150
200
250
300
0
2
4
6
8

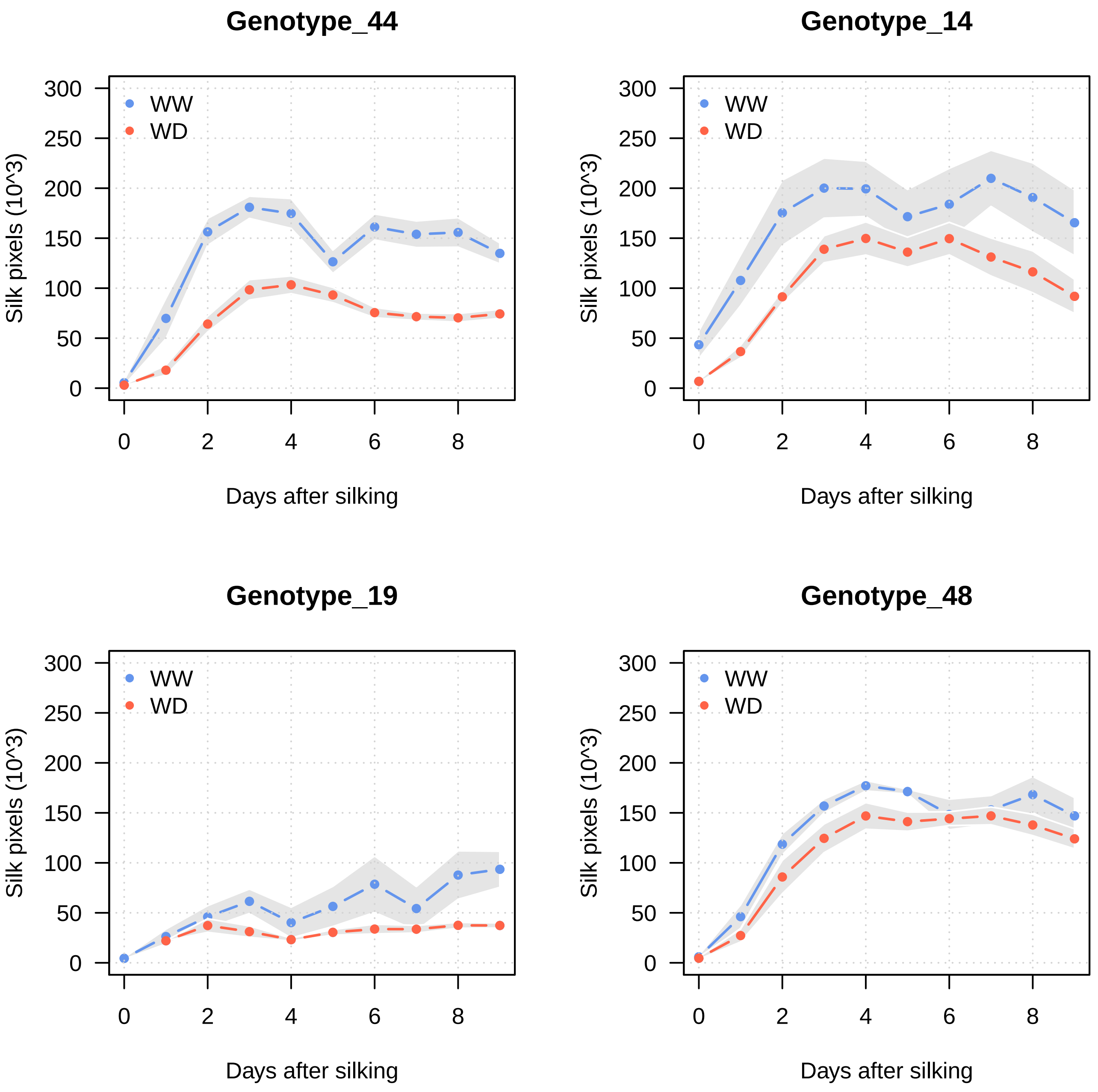
Genotype_44
Genotype_14
Genotype_19
Genotype_48
WW
WD
Silk pixels (10^3)
Days after silking
0
50
100
150
200
250
300
0
2
4
6
8

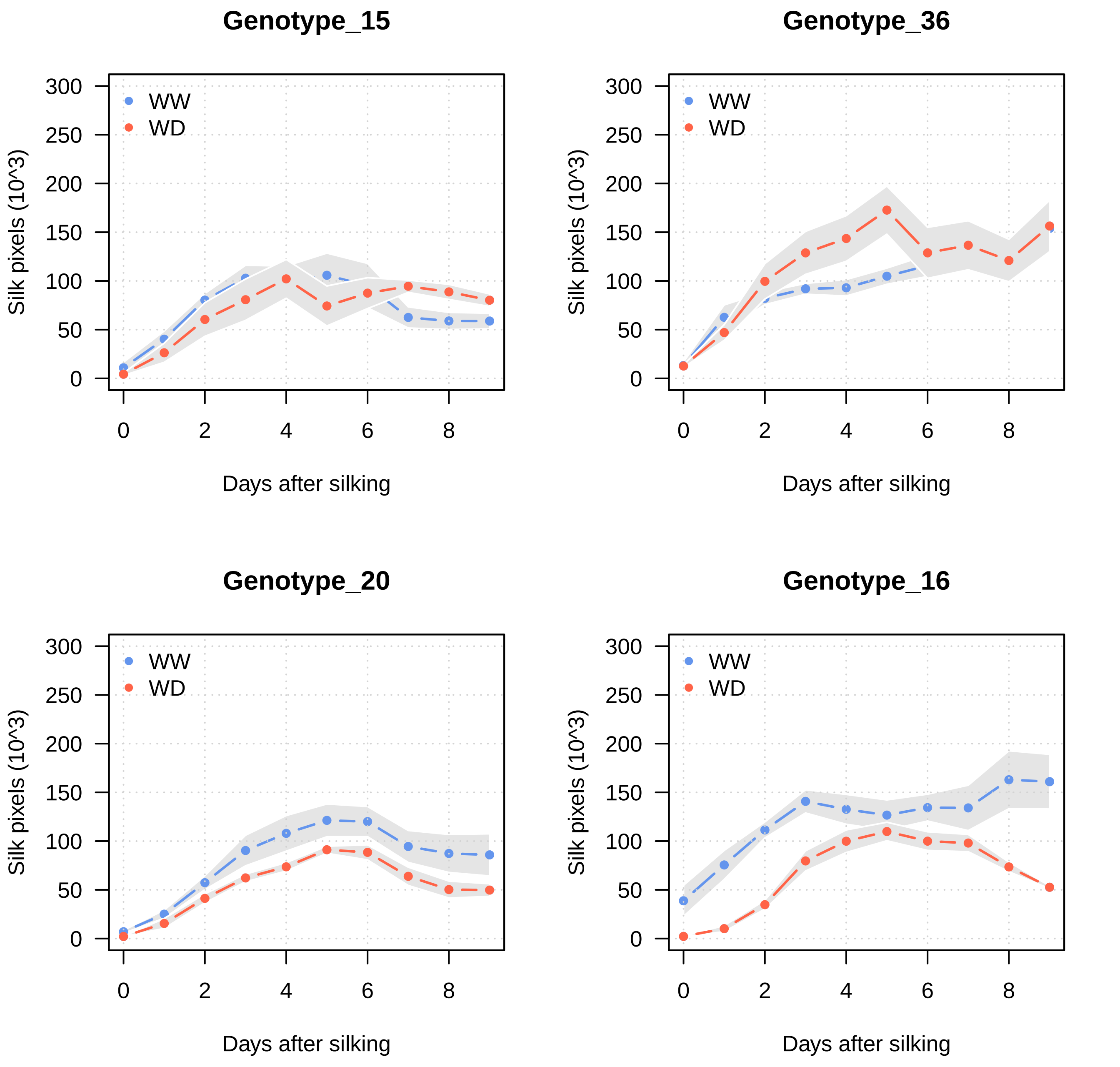
Genotype_15
Genotype_36
Genotype_20
Genotype_16
WW
WD
Silk pixels (10^3)
Days after silking
0
50
100
150
200
250
300
0
2
4
6
8

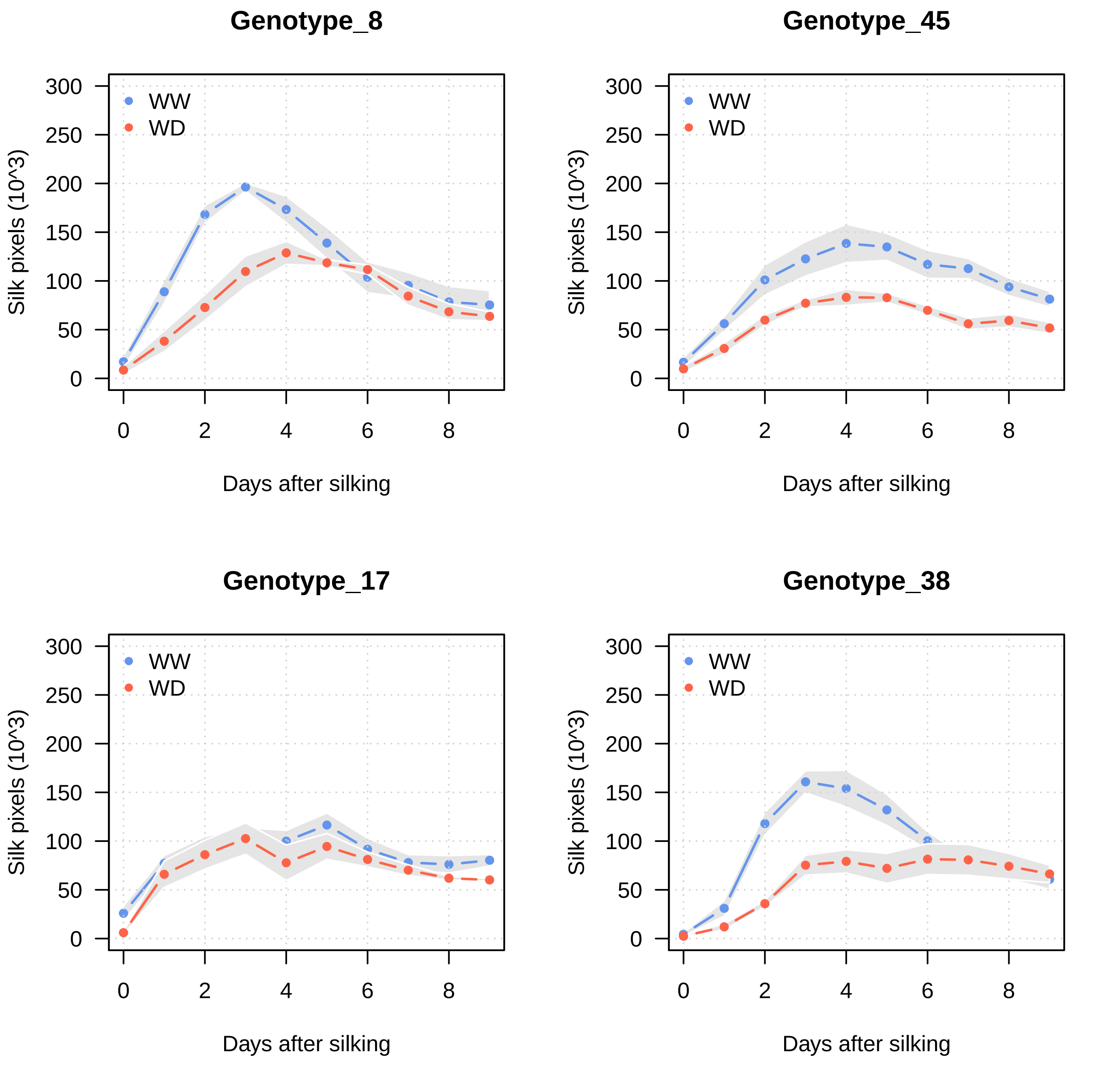
Genotype_8
Genotype_45
Genotype_17
Genotype_38
WW
WD
Silk pixels (10^3)
Days after silking
0
50
100
150
200
250
300
0
2
4
6
8

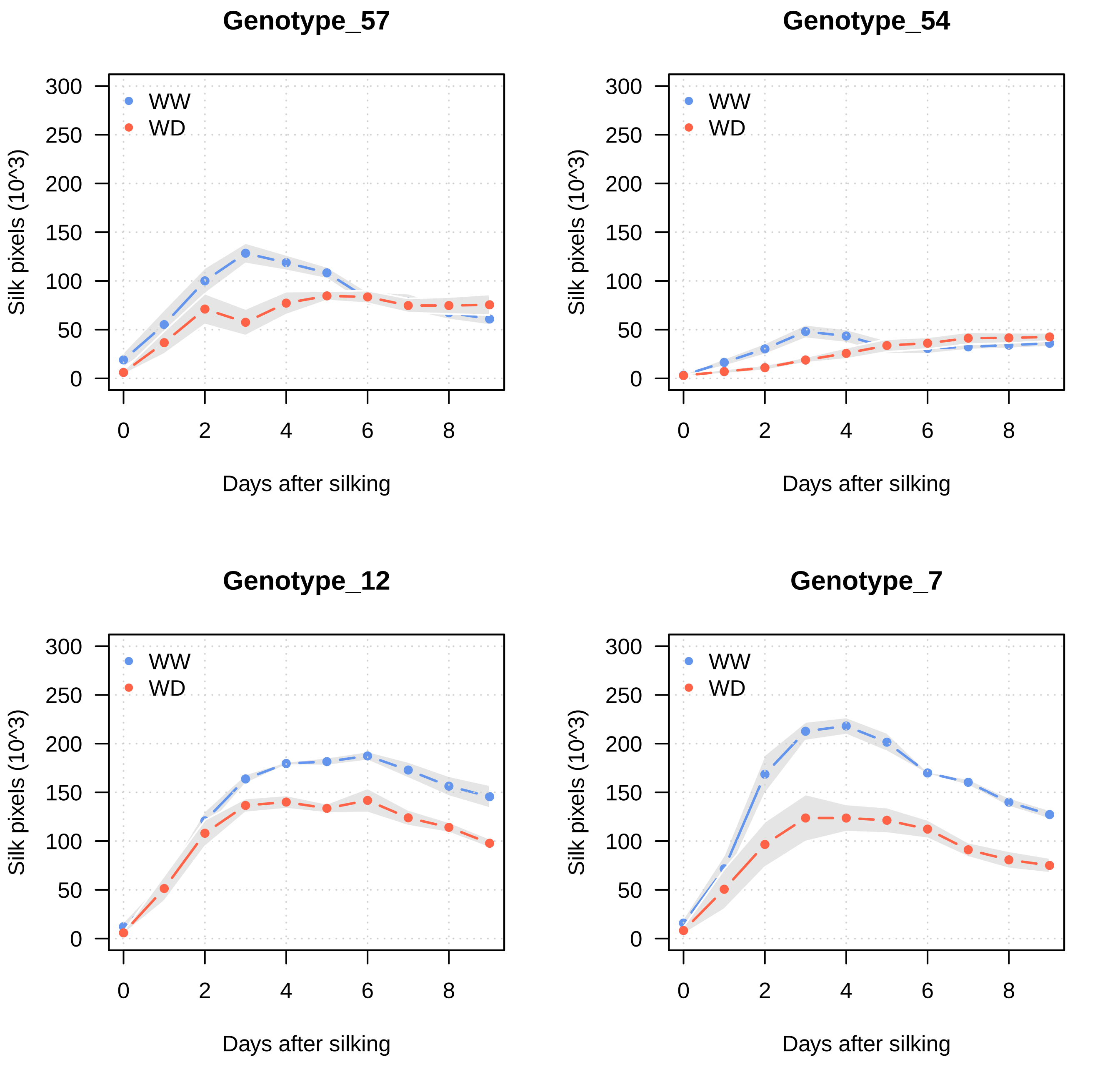
Genotype_57
Genotype_54
Genotype_12
Genotype_7
WW
WD
Silk pixels (10^3)
Days after silking
0
50
100
150
200
250
300
0
2
4
6
8

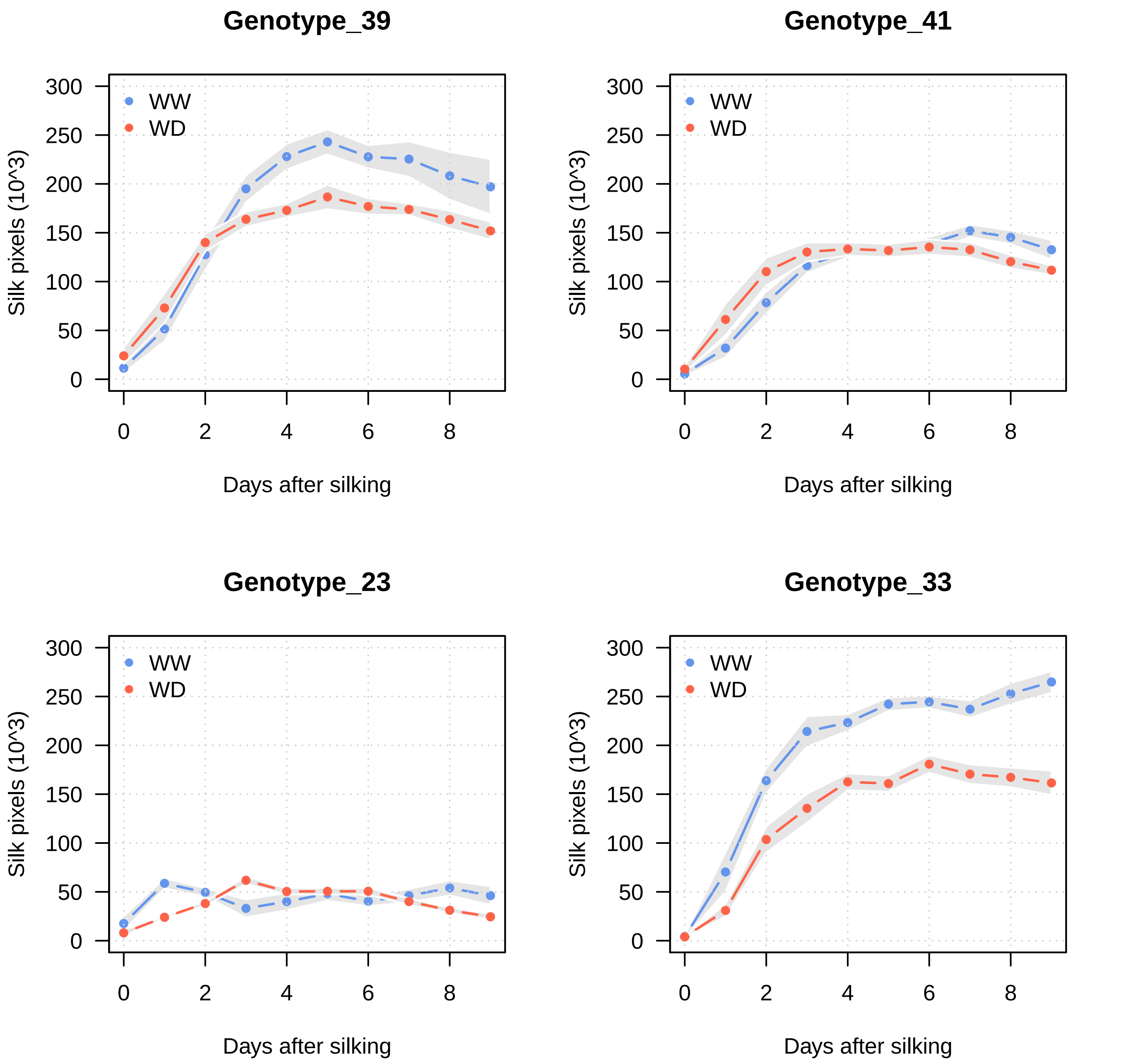

Genotype_39
Genotype_41
Genotype_23
Genotype_33
WW
WD
Silk pixels (10^3)
Days after silking
0
50
100
150
200
250
300
0
2
4
6
8

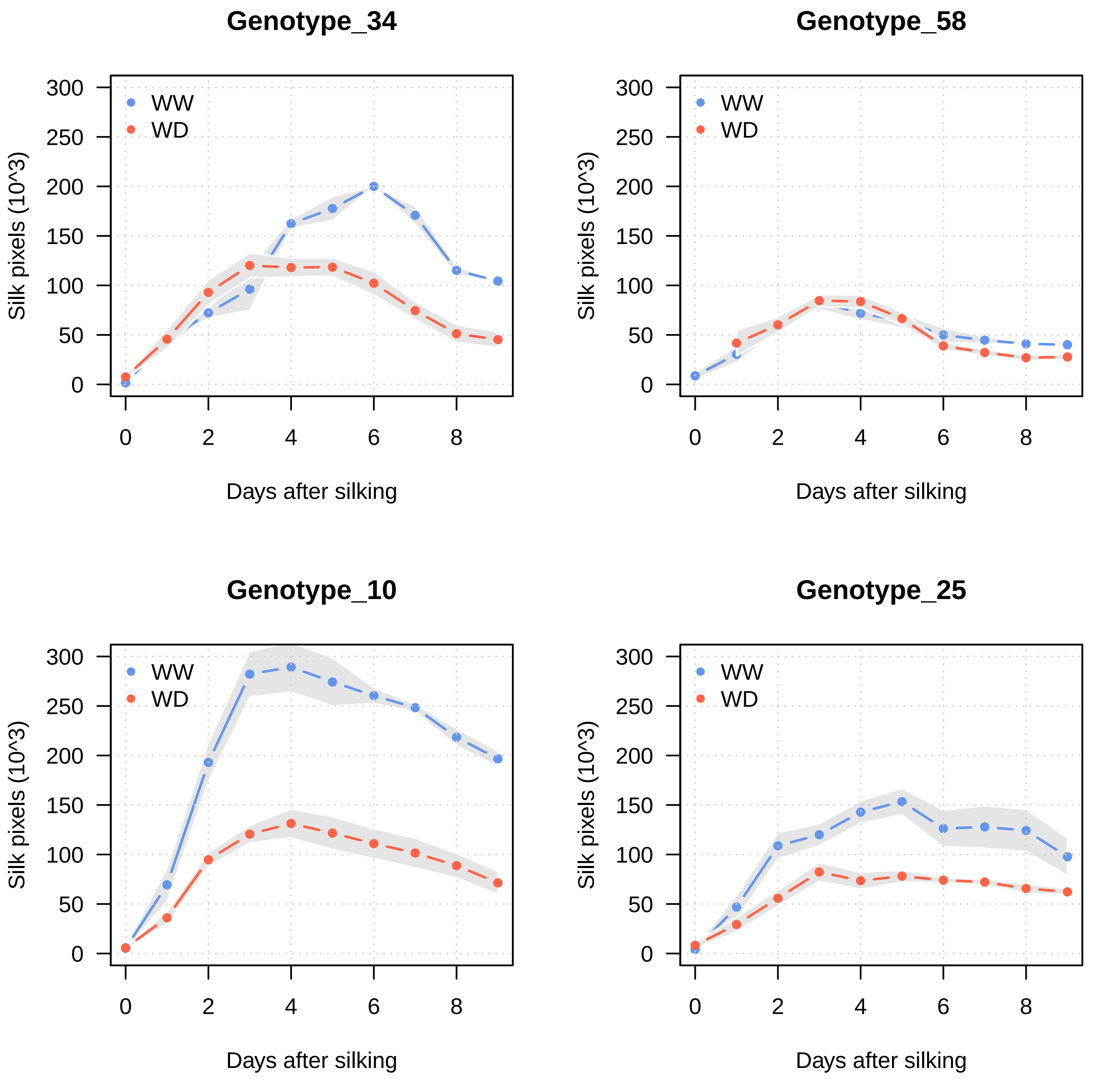
Genotype_34
Genotype_58
Genotype_10
Genotype_25
WW
WD
Silk pixels (10^3)
Days after silking
0
50
100
150
200
250
300
0
2
4
6
8

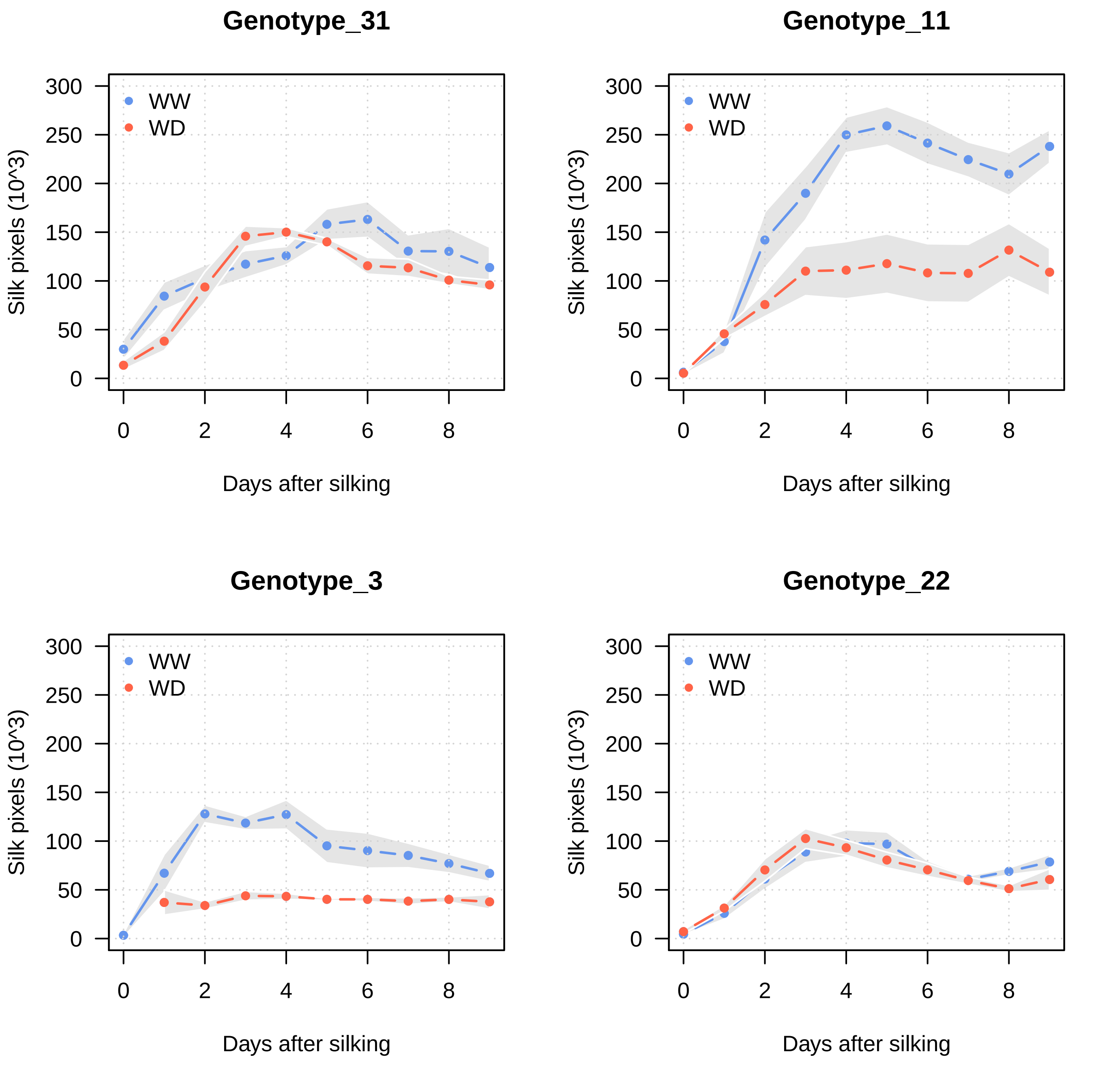
Genotype_31
Genotype_11
Genotype_3
Genotype_22
WW
WD
Silk pixels (10^3)
Days after silking
0
50
100
150
200
250
300
0
2
4
6
8

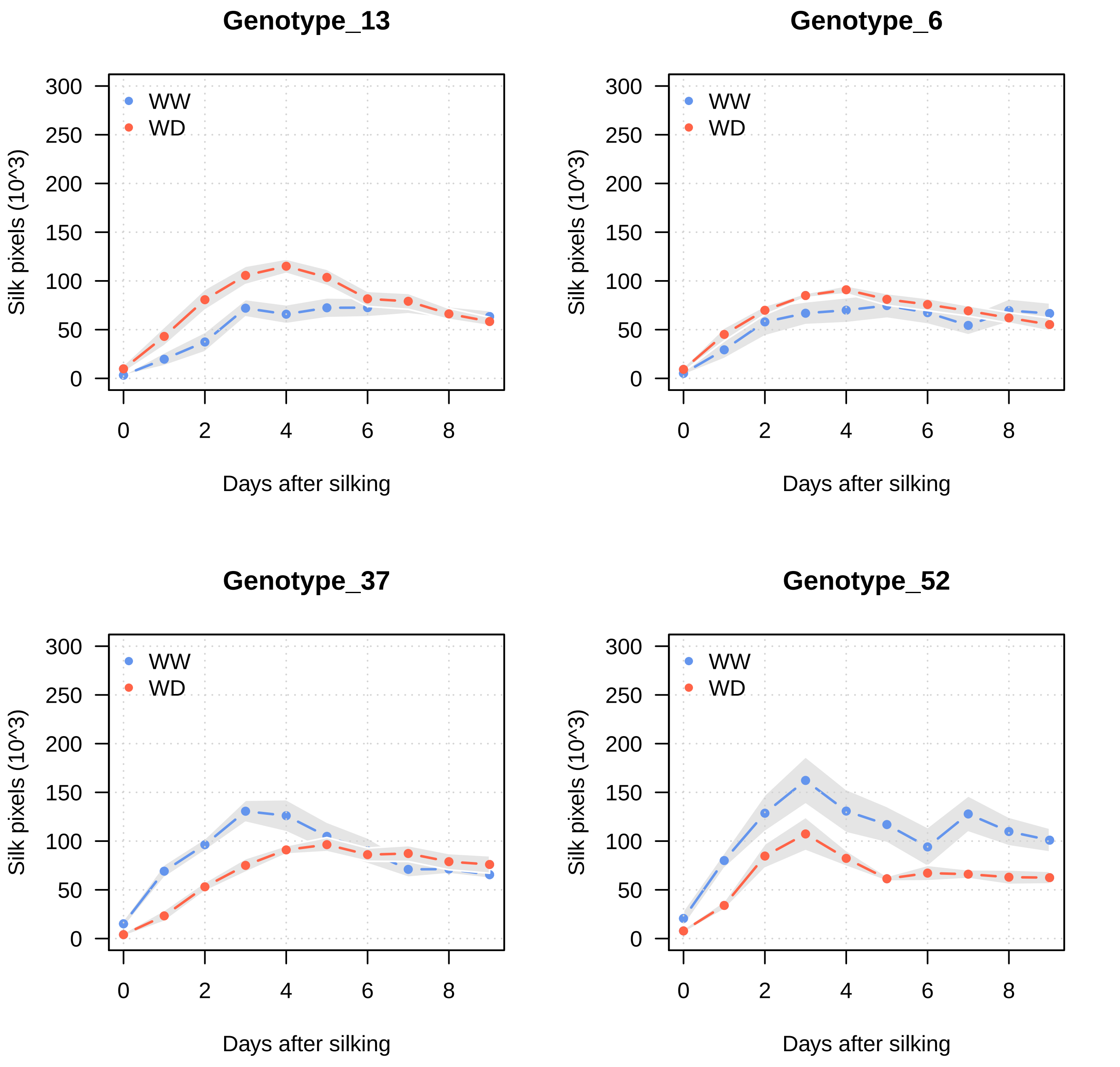
Genotype_13
Genotype_6
Genotype_37
Genotype_52
WW
WD
Silk pixels (10^3)
Days after silking
0
50
100
150
200
250
300
0
2
4
6
8

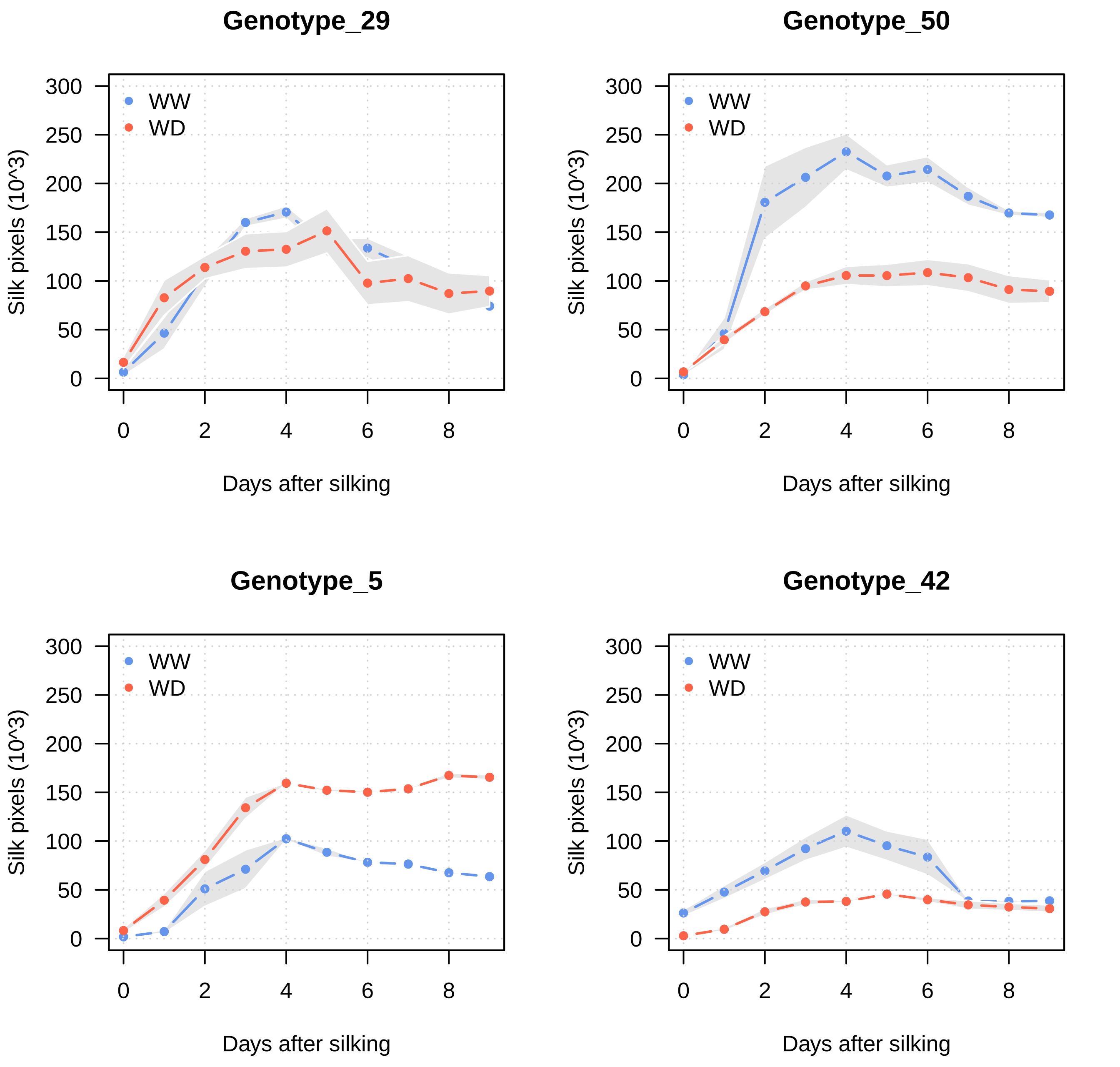
Genotype_29
Genotype_50
Genotype_5
Genotype_42
WW
WD
Silk pixels (10^3)
Days after silking
0
50
100
150
200
250
300
0
2
4
6
8
